# Sharpey-Schafer Lecture Gas channels

**DOI:** 10.1113/expphysiol.2010.055244

**Published:** 2010-09-17

**Authors:** Walter F Boron

**Affiliations:** Department of Physiology and Biophysics, Case Western Reserve University School of Medicine10900 Euclid Avenue, Cleveland, OH 44106-4970, USA

## Abstract

The traditional dogma has been that all gases diffuse through all membranes simply by dissolving in the lipid phase of the membrane. Although this mechanism may explain how most gases move through most membranes, it is now clear that some membranes have no demonstrable gas permeability, and that at least two families of membrane proteins, the aquaporins (AQPs) and the Rhesus (Rh) proteins, can each serve as pathways for the diffusion of both CO_2_ and NH_3_. The knockout of RhCG in the renal collecting duct leads to the predicted consequences in acid–base physiology, providing a clear-cut role for at least one gas channel in the normal physiology of mammals. In our laboratory, we have found that surface-pH (pH_S_) transients provide a sensitive approach for detecting CO_2_ and NH_3_ movement across the cell membranes of *Xenopus* oocytes. Using this approach, we have found that each tested AQP and Rh protein has its own characteristic CO_2_/NH_3_ permeability ratio, which provides the first demonstration of gas selectivity by a channel. Our preliminary AQP1 data suggest that all the NH_3_ and less than half of the CO_2_ move along with H_2_O through the four monomeric aquapores. The majority of CO_2_ takes an alternative route through AQP1, possibly the central pore at the four-fold axis of symmetry. Preliminary data with two Rh proteins, bacterial AmtB and human erythroid RhAG, suggest a similar story, with all the NH_3_ moving through the three monomeric NH_3_ pores and the CO_2_ taking a separate route, perhaps the central pore at the three-fold axis of symmetry. The movement of different gases via different pathways is likely to underlie the gas selectivity that these channels exhibit.

## Preface

This lecture honours Sir Edward Albert Sharpey-Schafer (1850–1935) and his grandson, Professor E. P. Sharpey-Schafer (1908–1963). The younger Sharpey-Schafer made important contributions to respiratory and cardiovascular physiology as a professor of medicine at St Thomas’ Hospital in London (Anon, 1963).

The elder Sharpey-Schafer, working in London and Edinburgh, was a pioneer in the field of endocrinology and a major figure in the public service of the discipline of physiology (Hill, 1935). He made the pioneering discovery that a ‘suprarenal extract’ (i.e. predominantly adrenaline) increases blood pressure (Oliver & Schafer, 1895). He coined or popularized the terms ‘endocrine’, ‘autocoid’ and ‘insuline’ (from the Latin *insula*= island); the last, after hypothesizing that the substance that regulates blood glucose emanates from the islets of Langerhans. Sharpey-Schafer's closest link to the subject of my lecture was his work in the field of ventilation, including the introduction of the ‘Schafer’ method of artificial respiration, a procedure in which one straddles at the hips a patient in the prone position, and then periodically applies pressure with both hands on the back over the lower ribs.

As a distinguished servant in the cause of physiology, Sir Edward A. Sharpey-Schafer was a founding member of the Physiological Society (1876), the editor of *Advanced Textbook of Physiology* (1898–99) and the founder and lead editor of *The Quarterly Journal of Experimental Physiology* (1908), the predecessor to the present journal, the first editorial board of which also included Gotch, Halliburton, Sherrington, Starling and Waller.

## Introduction: Overton's rule

**The work of Overton.** Over a century ago, Overton (Overton, 1897) performed a classic study on the algae *Spirogyra* in which he assessed the uptake of NH_3_ and various amines by monitoring the precipitation that occurred as the amines combined with naturally occurring tannins. He found that extracellular acidification, which converts NH_3_ to NH^+^_4_ and likewise converts primary, secondary and tertiary amines to their protonated/charged counterparts, reduces tannin precipitation. However, extracellular acidification had no effect in the case of quarternary amines, which are already positively charged. Overton concluded that it is the neutral weak base, rather than the cationic acidic form, that predominantly enters the cell. Overton subsequently studied the uptake of acids into frog muscle, using osmotic swelling to gauge solute influx. He found that neutral weak acids (e.g. acetic acid) were far more effective than more acidic solutions of strong acids, again leading him to conclude that the neutral species more easily crossed into cells, and supporting his insightful hypothesis that the cell membrane consists predominantly of lipids.

**Figure 1:**
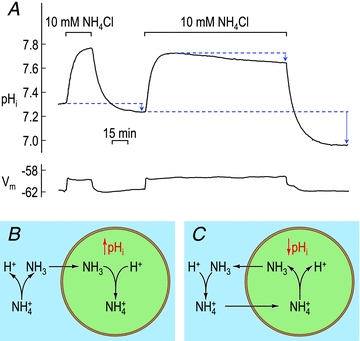
Effect of extracellular NH_3_/NH^+^_4_ on intracellular pH (pH_i_) of a squid giant axon, showing data (*A*), model of alkalinizing phase (*B*) and model of acidification during plateau phase (*C*) Throughout the experiment in *A*, artificial seawater (ASW) with an extracellular pH of 7.70 flowed past a cannulated axon, which was suspended in a chamber of small volume. Intracellular pH and membrane potential (*V*_m_) were monitored with glass microelectrodes. At the indicated times, the ASW was switched to one augmented with 10 mm NH_4_Cl. Data are from Boron & De Weer (1976*b*). *B* shows that the influx of NH_3_ leads to the consumption of intracellular H^+^ and thus a rise in pH_i_. This process accounts for the rising phase of pH_i_ during the two NH_3_/NH^+^_4_ exposures in *A*. The influx of NH_3_ in *B* leads to the dissociation of NH^+^_4_ near the extracellular surface of the membrane. In the bulk (i.e. flowing) ASW, the NH_3_/NH^+^_4_ buffer was in equilibrium (NH_3_+ H^+^⇌ NH^+^_4_). *C* shows the system after NH_3_ has equilibrated across the cell membrane; this equilibration corresponds to the peak pH_i_ during the second NH_3_/NH^+^_4_ pulse in *A*. After this equilibration, pH_i_ is dominated by the influx of NH^+^_4_; this NH^+^_4_ influx had been occurring since the beginning of the NH_3_/NH^+^_4_ exposure but its effect on pH_i_ had been overwhelmed by the influx of NH_3_. Now, during the plateau phase, the influx of NH^+^_4_ leads to a net dissociation of NH^+^_4_ in the cytosol. This process accounts for the plateau-phase acidification (i.e. falling phase of pH_i_) during the second NH_3_/NH^+^_4_ exposure in *A*. At the same time, the accumulation of NH_3_ inside the cell now leads to the net efflux of NH_3_, some of which consumes H^+^ on the outer surface of the cell, creating more NH^+^_4_ (the NH_3_/NH^+^_4_ shuttle). Because the cell accumulated NH^+^_4_ during the NH_3_/NH^+^_4_ exposure, the removal of extracellular NH_3_/NH^+^_4_ leads to a pH_i_ undershoot.

**Confirmation with NH_3_.** By studying cells containing native or exogenously applied pH-sensitive dyes, other investigators confirmed Overton's fundamental observations on a wide range of cell types. For example, several investigators showed that an exposure to NH_3_/NH^+^_4_ causes internal pH to rise (Warburg, 1910; Harvey, 1911; Jacobs, 1922). In more modern times, Roger Thomas in 1974 used a microelectrode to show that an exposure to NH_3_/NH^+^_4_ causes the intracellular pH (pH_i_) of a snail neuron to rise, and that the removal of extracellular NH_3_/NH^+^_4_ has the opposite effect (Thomas, 1974). In 1976, Boron and De Weer extended these observations in microelectrode experiments on squid axons (Boron & De Weer, 1976*b*), as illustrated by the twin-pulse experiment shown in Fig. 1*A*. In the brief, first exposure, pH_i_ rises monotonically as the entry of the weak base NH_3_ leads to the consumption of intracellular H^+^ and the formation of NH^+^_4_, as illustrated in Fig. 1*B*. After the removal of NH_3_/NH^+^_4_, the reactions in Fig. 1*B* reverse and pH_i_ falls but, curiously, to a value that modestly undershoots the initial pH_i_.

In the longer, second NH_3_/NH^+^_4_ exposure shown in Fig. 1*A*, one might have thought that pH_i_ should gradually approach an asymptote as [NH_3_]_i_ gradually approaches extracellular [NH_3_] or [NH_3_]_o_. Instead, pH_i_ rises to a peak and then begins a slower decline. This plateau-phase acidification is due to the entry of the weak acid NH^+^_4_. As shown in Fig. 1*C*, a small fraction of the entering NH^+^_4_ dissociates in the cytosol to form H^+^ and NH_3_. As [NH_3_]_i_ rises above [NH_3_]_o_, NH_3_ exits the cell and combines with extracellular H^+^ to form NH^+^_4_, which completes the cycle by entering the cell. Thus, during the plateau phase, NH_3_ effectively shuttles H^+^ into the cell. Even during the brief NH_3_/NH^+^_4_ exposure in Fig. 1*A*, the influx of NH^+^_4_, overwhelmed by the entry of NH_3_, must have slowed the rate of pH_i_ increase, reduced the magnitude of the overall NH_3_-induced pH_i_ increase and led to a modest, excess build up of intracellular NH^+^_4_. Upon removal of the extracellular NH_3_/NH^+^_4_, this excess intracellular NH^+^_4_ dissociates into NH_3_, which leaves the cell, plus H^+^, which causes the modest pH_i_ undershoot. In the longer NH_3_/NH^+^_4_ exposure, the much larger build up of intracellular NH^+^_4_ leads to a correspondingly greater pH_i_ undershoot.

The above work introduced the so-called ammonium-prepulse technique, which has become a widely used method for acid loading cells.

Depending on the cell type, a plateau-phase acidification like that in Fig. 1*A* can reflect the action of any of several mechanisms that acidify the cell. In barnacle muscle fibres, NH^+^_4_ entry through channels, presumably K^+^ channels, plays a major role (Boron, 1977; Kikeri *et al.* 1989). As discussed below, the Na^+^–K^+^ pump can take up NH^+^_4_, especially when [K^+^]_o_ is low (Aickin & Thomas, 1977), and the Na^+^–K^+^–2Cl^−^ cotransporter can also mediate a robust uptake of NH^+^_4_ (Kinne *et al.* 1986; Kikeri *et al.* 1989). In fact, because of similarities in the physicochemical properties of NH^+^_4_ and K^+^ in aqueous solution, any K^+^-transport pathway could be viewed as a potential means of NH^+^_4_ transport. Finally, in the presence of CO_2_/HCO^−^_3_, the Cl^−^–HCO^−^_3_ exchanger (a pH_i_-regulatory mechanism that is called into play when pH_i_ is too high) can make a contribution to the plateau-phase acidification (Vaughan-Jones, 1982). Regardless of the mechanism of the plateau-phase acidification, all work summarized above is consistent with Overton's view that the neutral weak base is the dominant species that moves through the membrane.

**Confirmation with CO_2_.** After Overton, Jacobs, using the native pH-sensitive dye in flower petals, confirmed Overton's results by demonstrating that the cells exposed to CO_2_/HCO^−^_3_ underwent a fall in internal pH (Jacobs, 1920). Caldwell, working on squid axons, was the first to observe a CO_2_-induced fall of pH_i_ using a pH-sensitive microelectrode (Caldwell, 1958), and Thomas, working on snail neurons with his newly designed glass microelectrode that was truly ‘micro’, was the first to show that the acidifying effect of CO_2_ is reversible (Thomas, 1974).

**Figure 2:**
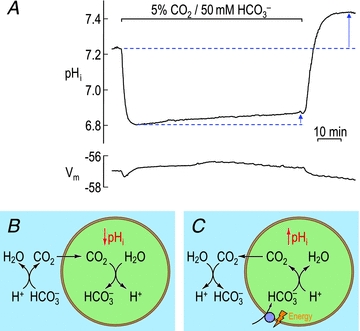
Effect of extracellular CO_2_/HCO^−^_3_ on intracellular pH of a squid giant axon, showing data (*A*), model of acidifying phase (*B*) and model of alkalinization during plateau phase (*C*) Throughout the experiment in *A*, ASW with an extracellular pH of 7.70 flowed past a cannulated axon, which was suspended in a chamber of small volume. Intracellular pH and membrane potential were monitored with glass microelectrodes. During the indicated period, the ASW was switched to one equilibrated with 5% CO_2_ and in which 50 mm NaHCO_3_ replaced 50 mm NaCl. Data are from Boron & De Weer (1976*b*). *B* shows that the influx of CO_2_ leads to the production of intracellular H^+^ and thus a fall in pH_i_. This process accounts for the falling phase of pH_i_ during the CO_2_/HCO^−^_3_ exposure in *A*. The influx of CO_2_ in *B* leads to the indicated reaction near the extracellular surface of the membrane. In the bulk (i.e. flowing) ASW, the CO_2_/HCO^−^_3_ buffer was in equilibrium (CO_2_+ H_2_O ⇌ H^+^+ HCO^−^_3_). *C* shows the system after CO_2_ has equilibrated across the cell membrane; this equilibration corresponds to the pH_i_ nadir during the CO_2_/HCO^−^_3_ pulse. After this equilibration, pH_i_ is dominated by ‘acid extrusion’, shown here as the active uptake of HCO^−^_3_. This active uptake of HCO^−^_3_ is mediated by a transporter called a Na^+^-driven Cl^−^–HCO^−^_3_ exchanger (which may mediate uptake of CO_3_^2−^ or NaCO_3_^−^ ion pair). This HCO^−^_3_ uptake had been occurring since the beginning of the CO_2_/HCO^−^_3_ exposure, but its effect on pH_i_ had been overwhelmed by the influx of CO_2_. Now, during the plateau phase, HCO^−^_3_ uptake leads to a consumption of H^+^ in the cytosol and thus the production of CO_2_, leading to a net efflux of CO_2_. This process accounts for the plateau-phase alkalinization (i.e. rising phase of pH_i_) during the CO_2_/HCO^−^_3_ exposure in *A*. Because the cell accumulated HCO^−^_3_ during the CO_2_/HCO^−^_3_ exposure, the removal of extracellular CO_2_/HCO^−^_3_ leads to a pH_i_ overshoot.

In their experiments on squid axons, Boron & De Weer (1976*b*) extended the earlier work by lengthening the time of the CO_2_/HCO^−^_3_ exposure. As shown in Fig. 2*A*, pH_i_ at first falls rapidly as the entry of CO_2_ leads to the formation of intracellular carbonic acid, which in turn dissociates to form intracellular H^+^ and HCO^−^_3_, as illustrated in Fig. 2*B*. Although one might expect that pH_i_ would gradually approach an asymptote as [CO_2_]_i_ gradually approaches [CO_2_]_o_, pH_i_ begins a slow increase that can only be explained by ‘acid extrusion’, the active removal of an acid (e.g. H^+^) or the active uptake of a base (e.g. HCO^−^_3_), as shown in Fig. 2*C*. Either way, acid extrusion would lead to the accumulation of excess HCO^−^_3_ inside the cell. With the subsequent removal of extracellular CO_2_/HCO^−^_3_, the excess intracellular HCO^−^_3_ combines with H^+^ and eventually exits the cell as CO_2_, producing the overshoot in Fig. 2*A*.

The experiment in Fig. 2*A* was the first example of the dynamic regulation of pH_i_. It had been surmised since the work of Fenn that, in the steady state, cells faced with the passive influx of H^+^ must extrude acid in order to maintain the observed pH_i_ (Fenn & Cobb, 1934; Fenn & Maurer, 1935). In 1975, Roos took the next important step in the field of pH_i_ regulation, when he exposed rat diaphragm muscle to either d-lactic acid (HLac ⇌ H^+^+ Lac^−^) or the weak acid DMO (HDMO ⇌ H^+^+ DMO^−^). He confirmed that, after a few hours, the cells had accumulated large amounts of d-lactate or DMO^−^, which implied, if the permeant species were HLac or HDMO, that the cytosol had undergone a massive acid load. Nevertheless, he found that the simultaneously computed pH_i_ was near the value of muscle fibres not so acid loaded. He correctly concluded that the cells, between the time of the acid load and the measurement of pH_i_, must have extruded the H^+^ load (Roos, 1975). The experiment in Fig. 2*A* directly demonstrated the sorts of processes that Roos had envisioned, and also revealed the time courses; a relatively rapid intracellular acid load, followed by a slower pH_i_ recovery due to an active process.

Later work in both the squid axon and the snail neuron demonstrated that the acid-extrusion mechanism in squid axons and snail neurons is due to a Na^+^-driven Cl^−^–HCO^−^_3_ exchanger (Boron & De Weer, 1976*a*; Russell & Boron, 1976; Thomas, 1976, 1977; Boron & Russell, 1983). In many other cells studied in the absence of CO_2_/HCO^−^_3_, the pH_i_ recovery from an acid load is mediated by a Na^+^–H^+^ exchanger, as first demonstrated by Aickin & Thomas (1977) for mouse skeletal muscle. The Na^+^–H^+^ exchanger had previously been demonstrated in membrane vesicles from small intestine and kidney by Murer *et al.* (1976), who approached the issue from the perspective of transepithelial transport. For a more in-depth treatment of the role of these transporters in pH_i_ regulation, the reader might consult reviews specifically on that topic (Roos & Boron, 1981; Boron, 2004; Bevensee & Boron, 2008; Vaughan-Jones *et al.* 2009; Casey *et al.* 2010).

**Cautionary notes on pH.** In his experiments, Overton measured the ability of an entering substance either to precipitate tannins or to cause osmotic swelling, both of which are reasonably direct measures of influx. The same cannot be said of the far more common, modern assays that exploit measurements of pH_i_ (e.g. Figs 1 and 2). These assays do not assess permeability *per se* but whether it is the neutral *versus* charged species of a buffer pair that has the dominant impact on pH_i_. However, conclusions in the literature are almost never stated in this limited manner. For example, the NH_3_-induced alkalinization in Fig. 1 does not prove that the cell membrane is impermeable to NH^+^_4_; in fact, the membrane is permeable to NH^+^_4_, which is the basis for the plateau-phase acidification in Fig. 1. In fact, such experiments do not even prove that the NH_3_ flux is greater than the NH^+^_4_ flux. As discussed in greater length elsewhere (Musa-Aziz *et al.* 2009*c*), one can conclude from Fig. 1 only that the ratio of the NH_3_ influx (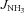) to the NH^+^_4_ influx (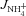) exceeds 10^(pHi − p*K*)^, a conclusion that flows from the analysis in the appendix of the paper by Boron & De Weer (1976*b*). For example, if pH_i_ is 7.3 and the p*K* of the NH_3_–NH^+^_4_ equilibrium is 9.3, exposing a cell to an NH_3_/NH^+^_4_ solution will cause pH_i_ to rise when:

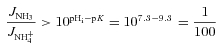
(1)In other words, even if the NH_3_ influx were only 1/99th of the NH^+^_4_ influx, pH_i_ would still rise, albeit slowly. Note that the limiting ratio of fluxes (1/100 in this case) does not translate directly to the limiting ratio of permeabilities. For the extracellular pH (pH_o_) values prevailing in most experiments on animal cells, [NH_3_]_o_ << [NH^+^_4_]_o_. For example, in the experiment of Fig. 1, pH_o_ was 8.0 and thus the ratio [NH_3_]_o_/[NH^+^_4_]_o_ was 1/20 or fivefold greater than the limiting ratio of fluxes. If we imagine that membrane potential (*V*_m_) were zero (so that we could ignore the effects of charge on the diffusion of NH^+^_4_), pH_i_ would rise as long as the permeability ratio 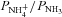 were <5. Thus, if 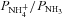 were, say, 4, then the 20-fold advantage in NH^+^_4_ concentration and fourfold advantage in NH^+^_4_ permeability would produce only an 80-fold advantage for the influx of NH^+^_4_ over NH_3_, which is still below the value of 100-fold necessary to stem the alkalinizing effect of NH_3_ on pH_i_.

We could use similar logic in analysing the CO_2_-induced acidification in Fig. 2. Here, one can conclude only that the ratio of CO_2_ influx (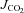) to HCO^−^_3_ influx (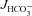) exceeds 10^(pHi − p*K*)^. Thus, if pH_i_ is 7.3 and the p*K* of the CO_2_–HCO^−^_3_ equilibrium is 6.1, exposing a cell to a CO_2_/HCO^−^_3_ solution will cause pH_i_ to fall as long as:

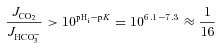
(2)Stated differently, even if the CO_2_ influx were only 1/15th of the HCO^−^_3_ influx, pH_i_ would still fall.

I should emphasize that I am not attempting here to challenge the dogma that membrane lipids are more permeable to electrically neutral species (e.g. NH_3_) than to their charged counterparts (e.g. NH^+^_4_); the dogma is true. However, I do point out that the prevalent pH_i_ data generally do not make as strong a case for Overton's conclusions as do Overton's original data.

**Overton's rule.** Although Overton's work provided important insights into the predominantly lipid nature of the cell membrane (see above), today Overton is remembered for ‘Overton's rule’. This principle, founded on the work of Overton and later investigators, states that membrane permeability to a substance X (*P*_X,m_) is proportional to the oil–water partition coefficient (*K*_f_) of X or, more precisely, the lipid–water partition coefficient of X (*K*_X_) for the lipid of the particular membrane under consideration. Thus, if *s*_X,aq_ is the solubility of X in an aqueous solution and *s*_X,m_ is the solubility in the membrane lipid, then *K*_X_=*s*_X,m_/*s*_X,aq_. We might regard Overton's rule as the solubility hypothesis, as follows:

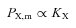
(3)Note that *P*_X,m_ is analogous to electrical conductance (reciprocal of resistance, *R*), and is only one determinant of the flux of X across the membrane (*J*_X_). If the concentration of X in the aqueous layer in contact with the extracellular or outer surface (oS) of the membrane is [X]_oS,aq_ and the concentration in the aqueous layer in contact with the intracellular or inner surface (iS) of the membrane is [X]_iS,aq_, then a simplified version of Fick's law yields the following:

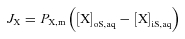
(4)In the next few paragraphs, we will focus on *P*_X,m_. However, almost never do physiologists measure *P*_X,m_ directly because they rarely have information about [X]_oS,aq_ or [X]_iS,aq_. Instead, physiologists generally measure the macroscopic permeability (*P*_X_) that governs the diffusion of X from the bulk (i.e. stirred) extracellular fluid that has a known concentration of X ([X]_o,bulk_), through the unstirred layer near the extracellular surface of the cell, through the membrane itself, and through an intracellular unstirred layer to some point (p) inside the cell where [X] is [X]_i,p_, as follows:

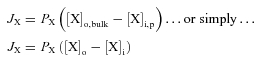
(5)Note that eqn (5) is highly oversimplified inasmuch as it ignores the following unstirred layers that envelope the cell membrane: (1) the extracellular unstirred layer (eUL) between the bulk extracellular fluid and the outer surface of the membrane; and (2) the intracellular unstirred layer (iUL) between the inner surface of the membrane and some point deeper inside the cytosol where we make our measurements.

Viewed differently, the overall ‘resistance’ that opposes the diffusion of X from the bulk extracellular fluid to a point in the intracellular fluid (*R*_X_= 1/*P*_X_) is the sum of the ‘resistance’ through the following: (1) the extracellular unstirred layer (*R*_X,eUL_= 1/*P*_X,eUL_); (2) the membrane (*R*_X,m_= 1/*P*_X,m_); and (3) the intracellular unstirred layer (*R*_X,iUL_= 1/*P*_X,iUL_). As is clear from the analysis of Pohl and colleagues (Missner *et al.* 2008*a*,*b*; Missner & Pohl, 2009), the combination of large unstirred layers (i.e. a relatively large sum *R*_X,eUL_+*R*_X,iUL_) and a relatively high *P*_X,m_ (i.e. a relatively small *R*_X,m_) renders *P*_X_ virtually insensitive to modest changes in *P*_X,m_. In other words, the permeability of the membrane *per se* only matters if *P*_X,m_ is relatively small compared with the aggregate permeability of the unstirred layers. We might term this the series-resistance problem, which we will consider again below (section ‘*A view from artificial bilayers*’).

**Consideration of the diffusion constant.** Over the decades, Overton's hypothesis evolved into Overton's rule. However, even as the rule became firmly cemented in our physiology textbooks, it became clear to the practitioners of membrane biology that the solubility hypothesis is overly simplistic. For example, many small molecules are more permeable than expected, with the increase being inversely related to molecular volume (Walter & Gutknecht, 1986). Biologists recognized that the permeability of substance X through the membrane depends not only on solubility but also the diffusion constant (*D*_X_). We might term this is the solubility-diffusion hypothesis (see Finkelstein, 1986), as follows:

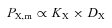
(6)The spirit of eqn (6) is clearly reflected in a physiology textbook with which I am intimately familiar (see Boron & Boulpaep, 2009), and others before it. In other words, after dissolving in the membrane lipid, X must diffuse across the membrane. For different solutes, *K*_X_ and *D*_X_ appear to weigh differently. In the case of CO_2_ transiting through artificial membranes, 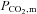 appears to be more sensitive to changes in *D*_X_ than *K*_X_. For example, CO_2_ solubility varies only about twofold among a wide range of lipids, whereas CO_2_ permeability has a range of over 1000 in artificial lipids (Blank & Roughton, 1960; Gutknecht *et al.* 1977; Simon & Gutknecht, 1980). Thus, 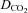 must be far more important a determinant of membrane permeability than 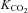, and some lipids must have far lower 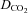 values than others.

Work exploiting electron paramagnetic resonance and O_2_-sensitive spin labels concludes that adding 50% cholesterol to a dimyristoyl phosphatidylcholine (DMPC) bilayer, by reducing the local product of [O_2_] and 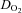 within the membrane, can reduce membrane O_2_ permeability by 75–80% of the value in a pure DMPC membrane (Subczynski & Swartz, 2005).

As an historical aside, chemists studying the diffusion of gases through polymers were, alas, well out in front of the physiologists. According to one review (Stannett, 1978), John Kearsley Mitchell had formulated what we now call ‘Overton's rule’ in 1831, well over a half century before Overton's experiments. The physical chemist Thomas Graham, who gave us Graham's law, discovered dialysis and is considered the founder of colloid chemistry, published his first paper on gas transport across membranes in 1829. He enunciated the solubility-diffusion theory in 1866, about a century before biologists.

**Consideration of integral membrane proteins.** Integral membrane proteins could reduce permeability by at least three general mechanisms.

First, it is important to recognize a trivial principle, that substances cannot dissolve in membrane lipid that is not there, having been displaced by integral membrane proteins that typically make up 25% of the membrane surface area. This figure is 50% or more in the erythrocyte (see Forster *et al.* 1998) and is presumably even higher in the membrane of the astrocytic end-foot that faces CNS vessels (these membranes consist of ∼35% aquaporin (AQP) 4 in semi-crystalline arrays; see Amiry-Moghaddam *et al.* 2004). A space-filling model of the synaptic vesicle (which identified only about half of the proteins) shows that the structure is ‘dominated’ by membrane proteins (see Fig. 4 of Takamori *et al.* 2006). Although a particular integral membrane protein may transport a restricted set of substances, we can generally regard any such protein as being an absolute barrier to most substances. An electron paramagnetic resonance study of O_2_ permeability suggests that, merely by displacing lipids, integral membrane proteins can reduce overall membrane permeability to half to a third of the value in an artificial lipid bilayer (Subczynski & Swartz, 2005).

A second mechanism by which integral membrane proteins can reduce permeability is by organizing the surrounding lipids (Engelman, 2005; Subczynski & Swartz, 2005; Subczynski *et al.* 2009). Electron paramagnetic resonance studies of O_2_ permeability indicate that integral membrane proteins reduce O_2_ permeability by creating slow oxygen transport (SLOT) domains in which O_2_ permeability can be reduced to 1/16 that of bulk-lipid domains in the same membrane (Kawasaki *et al.* 2001). At least in the example of the influenza virus membrane, the ratio of SLOT/bulk lipids is ∼40%/60%.

Together, mechanisms 1 and 2 reduce *K*_X_ and *D*_X_ to the effective values *K*′_X_ and *D*′_X_. We might term our updated model, which includes the ability of integral membrane proteins to displace and organize lipids, the solubility-diffusion-protein hypothesis, expressed as follows:

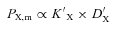
(7)The third mechanism by which integral membrane proteins can reduce permeability is by contributing to the reduction of access/egress as discussed in the following section.

**Consideration of access to and egress from the membrane lipid.** In addition to solubility, diffusion and integral membrane proteins, I would add a third consideration, that solubilization and diffusion (as modulated by integral membrane proteins) are only possible after the substance has gained access to the membrane lipid, and can continue only if the solute can exit the membrane. We might term this the access-solubility-diffusion-protein-egress hypothesis, as follows:

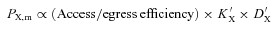
(8)Access/egress efficiency is almost certainly not 100%. Integral membrane proteins presumably reduce access/egress by at least two mechanisms. First, as pointed out by Engelman, even integral membrane proteins with modest cross-sectional areas in the plane of the lipid bilayer can have impressive ‘ectodomains covering lipid and creating steric restrictions’ (Engelman, 2005). Second, integral membrane proteins can form complexes with soluble proteins. As in the case of the large ectodomains, if a soluble-but-bound protein literally abuts the lipid, it insulates the lipid surface from the aqueous solution. If the protein hovers some distance from the membrane, it restricts diffusion by increasing the tortuosity factor between the bulk fluid and the lipid surface.

Some soluble proteins can attach to the membrane lipid, independent of integral membrane proteins, via ionic or hydrophobic interactions. These attached proteins, and other soluble proteins that attach to them, could restrict access/egrees to/from membrane lipids as outlined above for the ectodomains of integral membrane proteins and for soluble proteins adhering to these ectodomains. The plasma membrane, particularly the inner surface with its phosphatidyl serine (Subczynski & Swartz, 2005) and phosphoinositides, is the major locus of the cell's negative membrane-surface charge and strongly attracts soluble polycationic proteins (see Leventis & Grinstein, 2010).

Even those phospholipid head groups not masked by proteins can locally organize water molecules and thereby create an energy barrier to CO_2_ entry into/exit from lipid membranes (Wang *et al.* 2007). Sugar polymers attached to the outer surface of the plasma membrane could also reduce access to membrane lipid.

**Overall effect on background membrane permeability.** It may be worth noting that, whereas *D*_X_ is a kinetic term that describes the rate of diffusion, the lipid–water partition coefficient is a thermodynamic term that describes, at equilibrium, the concentration ratio of substance X in membrane lipid to water. The term *K*_X_ says nothing about the speed with which X reaches its equilibrium concentration in membrane lipid. Thus, *K*_X_ does not define the concentration of X at any distance through the membrane lipid, but the maximal possible [X] at infinite time with equal [X] on opposite sides of the membrane. While X is entering a cell, for example, the [X] at any distance through the thickness of the membrane lipid (i.e. around proteins in the plane of the membrane) depends on the following: (1) [X] in the aqueous layer near the extracellular surface of the membrane; (2) access efficiency; (3) the kinetics of solubilisation; (4) the effective *K*_X_ (which determines the upper bound of [X] in lipid) as reduced by integral membrane proteins; (5) the effective diffusion constant within the membrane lipid as reduced by integral membrane proteins; (6) the kinetics of desolubilization; (7) egress efficiency; and (8) [X] in the aqueous layer near the intracellular surface of the membrane. In other words, the solubility hypothesis (i.e. Overton's rule) merely sets an upper bound on the permeability properties of the lipid portion of the membrane, and cannot predict how far below this theoretical maximum the permeability may be in the lipid phase of a real biological membrane in various physiological conditions.

By how much might the presence of integral membrane proteins and the presence of cholesterol in bulk membrane lipids reduce ‘background’ membrane permeability? If proteins occupied two-thirds of the membrane surface, if 40% of the lipids were protein associated (assumed 1/16 of normal lipid permeability), and if 60% of the lipids had a 50% molar ratio of cholesterol (assumed 1/5 of normal lipid permeability), then the background permeability might fall to ∼5% of the nominal value. Reduced access/egress efficiency caused by ectodomains of integral membrane proteins and by adherent soluble proteins could further reduce this figure.

## Chinks in Overton's armour

Despite the cautionary notes in the previous section, in the early 1990s I did not know anyone, including me, who questioned Overton's rule, or the implicit dogma that all gases move through all membranes simply by dissolving in the lipid phase of the membrane. According to this philosophy, gas transport depends only on concentration gradients and the properties of the lipid phase of the membrane, leaving no possibility of regulation, and little possibility of selectivity beyond what might be allowed by solubility-diffusion theory. But then things began to change. …

**Membranes with relatively low NH_3_/NH^+^_4_ permeability ratios.**Hamm *et al.* (1985) demonstrated that the apparent transepithelial NH_3_ permeability of the isolated, perfused cortical collecting tubule is much lower than for proximal convoluted tubules (∼5 × 10^−3^*versus*∼6 × 10^−2^ cm s^−1^), which was perhaps the first argument consistent with restricted NH_3_ permeation. However, sceptics might argue that the difference could reflect the much higher surface area of proximal-tubule cells.

Garvin *et al.* (1988) examined the transepithelial permeability of NH_3_*versus* NH^+^_4_ in renal thick ascending limb (TAL). This nephron segment is peculiar, and important, because its apical membrane (i.e. the one facing the lumen) has a very low permeability to H_2_O. Thus, the reabsorption (i.e. movement from lumen to blood) of NaCl by the TAL is disproportionately high compared with the reabsorption of H_2_O, leaving behind in the lumen a relative surplus of H_2_O (hence, the term ‘diluting segment’) and simultaneously creating a hypertonic interstitium. The TAL also plays a critical role in transferring NH^+^_4_ from the lumen to the interstitium and then short-circuiting it to the collecting ducts for excretion in the urine. Garvin and colleagues found that the apparent transepithelial NH_3_ permeability of the TAL, like that of the cortical collecting tubule, is quite low (∼3.1 × 10^−3^ cm s^−1^). However, more telling was the observation that this value was only about 20-fold greater than the transepithelial NH^+^_4_ permeability (∼1.5 × 10^−4^ cm s^−1^), far lower than one would predict by Overton's rule. These data are consistent with the hypothesis that the TAL epithelium either restricts the diffusion of NH_3_ and/or enhances the transport of NH^+^_4_ via channels/transporters.

Kikeri *et al.* (1989) extended the work of Garvin and colleagues by monitoring the pH_i_ of TAL cells while introducing NH_3_/NH^+^_4_ to the lumen. Rather than the usual initial rise in pH_i_, they observed only a sustained fall. Aickin & Thomas (1977) had observed a large and sustained acidification in the presence of extracellular NH_3_/NH^+^_4_, but only after replacing extracellular K^+^ with NH^+^_4_ (presumably forcing the Na^+^–K^+^ pump to carry NH^+^_4_, rather than K^+^, into the cell), and even then they sometimes observed a small transient rise in pH_i_ (due to NH_3_ entry). Thus, Kikeri and coworkers demonstrated for the first time, in more-or-less physiological conditions, that the effects of NH^+^_4_ influx can overwhelm those of NH_3_ influx from the perspective of pH_i_. By analogy with eqn (1), we can conclude the following:

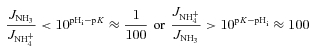
(9)These inequalities are consistent with the earlier data of Garvin *et al.* (1988). Despite the title of the Kikeri paper, one cannot really conclude from the data that the apical membrane of the TAL cells is impermeable to NH_3_, only that the flux of NH^+^_4_, carried by apical Na^+^–K^+^–2Cl^−^ cotransporters and K^+^ channels, greatly dominates over that of NH_3_ from the perspective of pH_i_.

**A membrane with no detectable permeability to NH_3_ or NH^+^_4_.** In 1989, surgical resident Steven Waisbren approached me with the idea of studying pH_i_ regulation in gastric parietal cells. The initial suggestion was to dissociate these cells from gastric glands and study them in isolation. I remember my almost reflex-like response, ‘Not in my lab!’… with the explanation that these are epithelial cells and it is important to respect their sidedness. This instinct proved to be critical. We decided to hand-dissect single glands from the fundus of a rabbit stomach (Waisbren *et al.* 1994*a*) and to perfuse the isolated gland as one would a renal tubule (Burg *et al.* 1966). Waisbren sucked up the blind end of a rabbit gland into a pipette assembly and pierced the base of the gland with the perfusion pipette, thereby initiating perfusion in the orthograde direction. The challenge is that the gastric-gland lumen is not so much the inside of a garden hose as it is a twisting ribbon.

Waisbren's first goal was to acid load the cells using an NH^+^_4_ prepulse (see Fig. 1) and then examine the pH_i_ recovery from the acute acid load. Owing to the plumbing of the perfusion-pipette system, the user-initiated switching of luminal solutions entails a lengthy (e.g. ∼20 s) and somewhat variable delay before the new solution (in this case, the one containing 20 mm NH_3_/NH^+^_4_) arrives in the lumen. Thus, it was our practice when working with proximal tubules first to switch the luminal solution, wait for pH_i_ to begin to rise (indicating arrival of NH_3_ in the lumen), and then to switch the basolateral or ‘bath’ solution (which arrives with predictable rapidity). Employing this protocol with a gastric gland, Waisbren switched the luminal solution from our standard Hepes-buffered saline at pH 7.40 to an otherwise identical solution in which he replaced 20 mm NaCl with 20 mm NH_3_/NH^+^_4_ and he waited … and waited for pH_i_ to rise.

**Figure 3:**
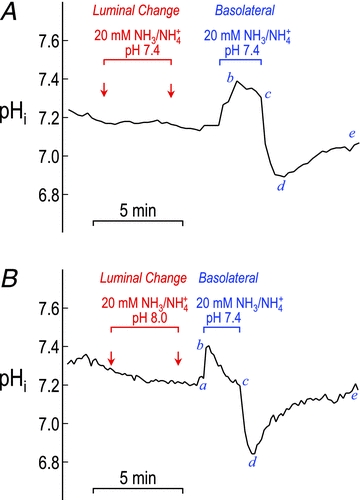
Effect of luminal *versus* basolateral NH_3_/NH^+^_4_ on intracellular pH of parietal cells of isolated, perfused gastric glands, with a luminal pH of 7.4 (*A*) and 8.0 (*B*) Throughout the experiment, the lumen of the gland was perfused, and the basolateral surface (‘bath’) was superfused with CO_2_/HCO^−^_3_-free physiological saline at 37°C. Intracellular pH of multiple parietal and chief cells was measured using the pH-sensitive dye BCECF in conjunction with a digital-imaging system. Data are from Waisbren *et al.* (1994*b*); similar data were obtained on chief cells. During the indicated periods, either the luminal or the basolateral solution was switched to one in which 20 mm NH_4_Cl replaced 20 mm NaCl. In *A*, both luminal and basolateral NH_3_/NH^+^_4_ solutions had a pH of 7.4. In *B*, the luminal NH_3_/NH^+^_4_ solution had a pH of 8.0 (and thus fourfold higher [NH_3_]), whereas the basolateral NH_3_/NH^+^_4_ solution had a pH of 7.4. The basolateral NH_3_/NH^+^_4_ exposures produced pH_i_ transients (*abcd*) similar to that in the second NH_3_/NH^+^_4_ pulse in Fig. 1*A*, except that here the pH_i_ recovered from the acid load (*de*). However, the luminal exposures produced no significant pH_i_ changes. Together with other data, these observations showed that the apical membranes of parietal and chief cells have no detectable permeability to either NH_3_ or NH^+^_4_.

Waisbren called to me several minutes after he had initiated that luminal solution switch, and announced the unexpected null result. We then watched together as he switched the basolateral solution to one containing 20 mm NH_3_/NH^+^_4_, and observed the ‘usual’ series of pH_i_ changes for an ‘NH^+^_4_ prepulse’. Figure 3*A* shows a parietal-cell pH_i_ record from such an experiment (Waisbren *et al.* 1994*b*). Note that, during the first part of the experiment, luminal [NH_3_] was ∼0.40 mm (at pH 7.4, [NH_3_]/[NH^+^_4_]= 0.4/19.6 ≅ 0.02) but did not cause a change in pH_i_. However, the same solution applied to the bath elicited pH_i_ transients typical of an NH^+^_4_ prepulse (segments *abcde*). Figure 3*B* shows a similar experiment, but one in which—during the exposure to 20 mm luminal NH_3_/NH^+^_4_—the luminal pH was 8.00 (rather than 7.40). Thus, luminal [NH_3_] was ∼1.46 mm ([NH_3_]/[NH^+^_4_]= 1.46/18.54 ≈ 0.08). Nevertheless, although the [NH_3_]/[NH^+^_4_] ratio was about fourfold higher than in Fig. 3*A*, pH_i_ still did not change during the luminal exposure to NH_3_/NH^+^_4_. In still other experiments (not shown), Waisbren replaced all 135 mm luminal Na^+^ with 135 mm NH_3_/NH^+^_4_ at pH 7.4 ([NH_3_]/[NH^+^_4_]≈ 0.08 but at much higher [NH_3_] and [NH^+^_4_] values than in Fig. 3*A*) but pH_i_ still did not budge. He obtained similar results from gastric chief cells.

Assuming that the above experiments were technically correct, can we explain the absence of a luminal-NH_3_/NH^+^_4_-induced pH_i_ change on the basis of serendipitous combinations of NH_3_ and NH^+^_4_ influxes across the apical membrane? We need only consider the case of parallel influxes of NH_3_ and NH^+^_4_. Parallel effluxes are probably impossible, given the absence of both NH_3_ and NH^+^_4_ in the basolateral solution. Fluxes of NH_3_ and NH^+^_4_ in opposing directions would always produce a pH_i_ change, with pH_i_ falling with an NH_3_ influx and rising with an NH_3_ efflux (Boron & De Weer, 1976*b*). By analogy with eqn (1), and as discussed elsewhere (Musa-Aziz *et al.* 2009*c*), we can define a ratio of NH_3_ and NH^+^_4_ fluxes that would produce no change in pH_i_ as follows:

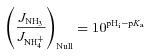
(10)Even if the condition prescribed in eqn (10) (that is, 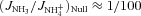) were satisfied in Fig. 3*A* when luminal (L) pH was 7.4 and luminal [NH_3_]/[NH^+^_4_] was ∼0.02, is it reasonable to expect the same equation to be satisfied in Fig. 3*B* when pH_L_ was 8.0 and [NH_3_]_L_/[NH^+^_4_]_L_ was ∼0.08? In the latter case, [NH_3_]_L_ was about fourfold higher and [NH^+^_4_]_L_ was somewhat reduced. Moreover, in the case with 135 mm NH_3_/NH^+^_4_ in the lumen, we would have expected NH^+^_4_ transport through a transporter or a channel eventually to saturate and thus shift (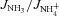) away from 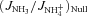. Thus, it is difficult to escape the conclusion that the apical membranes of both parietal and chief cells, exposed to perhaps the most hostile environment in the body, have an undetectably low permeability to both NH^+^_4_ and NH_3_.

**Figure 4:**
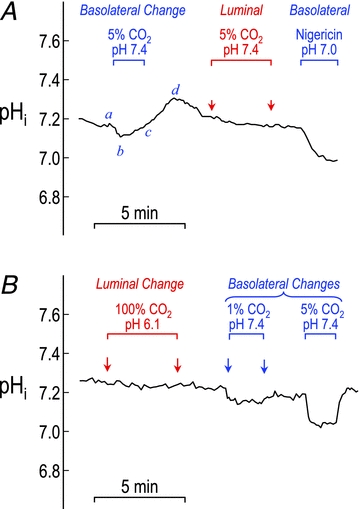
Effect of luminal *versus* basolateral CO_2_/HCO^−^_3_ on intracellular pH of parietal cells of isolated, perfused gastric glands, with a luminal pH of 7.4 (*A*) and 6.1 (*B*) Throughout the experiment, the lumen of the gland was perfused, and the basolateral surface (‘bath’) was superfused with physiological saline at 37°C. Intracellular pH of multiple parietal and chief cells was measured using the pH-sensitive dye BCECF in conjunction with a digital-imaging system. Data are from Waisbren *et al.* (1994*b*); similar data were obtained on chief cells. During the indicated periods, either the luminal or the basolateral solution was switched to one equilibrated with CO_2_. In *A*, both luminal and basolateral CO_2_/HCO^−^_3_ solutions had a pH of 7.4 achieved with 22 mm HCO^−^_3_. The basolateral CO_2_/HCO^−^_3_ exposures produced pH_i_ transients (*abcd*) similar to that in Fig. 2*A*. However, the luminal exposure produced no significant pH_i_ changes. This experiment terminated with a nigericin calibration. In *B*, the luminal CO_2_/HCO^−^_3_ solution had a pH of 6.1 (with 100% CO_2_/22 mm HCO^−^_3_) but produced no significant pH_i_ change. The basolateral CO_2_/HCO^−^_3_ solutions had pH values of 7.4 (1% CO_2_/4.4 mm HCO^−^_3_ or 5% CO_2_/22 mm HCO^−^_3_) and produced the expected acidifications. Basolateral 200 μm DIDS blocked the pH_i_ recovery from the acid loads. Together with other data, these observations showed that the apical membranes of parietal and chief cells have no detectable permeability to either CO_2_ or HCO^−^_3_.

**A membrane with no detectable permeability to CO_2_ or HCO^−^_3_.** In the same study as that discussed above in conjunction with Fig. 3, Waisbren examined the effects of exposing the apical and basolateral membranes to CO_2_/HCO^−^_3_ (Waisbren *et al.* 1994*b*). The initial portion of Fig. 4*A* shows that a basolateral exposure to 5% CO_2_/22 mm HCO^−^_3_ (pH 7.40) produces a rapid CO_2_-induced fall in pH_i_ (segment *ab*) followed by a slower recovery (segment *bc*) that reflects the pH_i_-regulatory activity of this gastric parietal cell. The removal of the basolateral CO_2_/HCO^−^_3_ causes a pH_i_ increase (segment *cd*) due to CO_2_ efflux followed by a pH_i_ relaxation (following segment *d*). A subsequent exposure to luminal 5% CO_2_/22 mm HCO^−^_3_ at the same pH of 7.40 had no effect on pH_i_. Figure 4*B* shows an experiment in which Waisbren blocked pH_i_ regulation with 200 μm 4,4′-diisothiocyanatostibene-2,2′-disulfonic acid (DIDS) in order to detect small CO_2_-induced pH_i_ decreases more easily. His first manoeuvre, introducing 100% CO_2_/22 mm HCO^−^_3_ (pH ∼6.1) into the lumen, had no effect on pH_i_. Nevertheless, subsequent basolateral exposures to 1% CO_2_/HCO^−^_3_ and 5% CO_2_/HCO^−^_3_ produced graded CO_2_-induced decreases in pH_i_ but no pH_i_ recovery. Waisbren obtained similar results on chief cells. Comparable to the NH_3_/NH^+^_4_ data, luminal CO_2_ did not acidify cells even though the CO_2_/HCO^−^_3_ ratio varied by a factor of ∼20 between Fig. 4*A* and *B*. Thus, it is not clear how a fortuitous combination of CO_2_ and HCO^−^_3_ influxes could have generated a null pH_i_ effect in both conditions. Calculations show that, ignoring permeability to HCO^−^_3_, the CO_2_-permeability × area product of the apical membrane could be no more than 1/1000 that of the basolateral membrane. We concluded that the apical membranes of gastric parietal and chief cells have no detectable permeability to either CO_2_ or HCO^−^_3_.

**Control experiments for the gastric-gland study.** The above data contain several control experiments that make it unlikely that we inadvertently failed to detect a luminal CO_2_-induced pH_i_ decrease that was in fact there. Nevertheless, one might still argue that the permeability of the apical membranes is so high that all available NH_3_ or CO_2_ diffused into the gastric-gland cells in the first few micrometres of the perfused gland lumen, leaving little to enter the cells over the bulk of the gland. However, we found that all cells in the perfused gland behaved in a similar fashion. Moreover, when we perfused the lumen with an unbuffered solution containing a pH-sensitive dye, switching the luminal perfusate from 5% CO_2_/22 mm HCO^−^_3_ (pH 7.4) to 100% CO_2_/HCO^−^_3_ (pH 6.1) caused an abrupt fall in luminal pH along the entire gland. If CO_2_ had been exiting the lumen, pH_L_ would have become gradually more alkaline at increasing distance from the perfusion pipette. Thus, we can conclude that, although the basolateral membranes of gastric glands have normal NH_3_/NH^+^_4_ and CO_2_/HCO^−^_3_ transport properties, the apical membranes have no detectable permeability to NH_3_ or NH^+^_4_, or to CO_2_ or HCO^−^_3_, making these the first documented gas-impermeable membranes within our limits of detection.

**The apical membrane of colonic crypts.** In 1995, Gastrointestinal Fellow Satish Singh published a pH_i_ study demonstrating that, like the cells of the rabbit gastric gland, those of the colonic crypt exhibit the normal sequence of pH_i_ changes when exposed to basolateral NH_3_/NH^+^_4_, but show no evidence of NH_3_ or NH^+^_4_ permeability at the apical membrane (Singh *et al.* 1995). Particularly striking was a comparison of 4 mm basolateral NH_3_/NH^+^_4_ at pH 7.4 ([NH_3_]/[NH^+^_4_]≈ 0.02) *versus* 100 mm luminal NH_3_/NH^+^_4_ at pH 8.0 ([NH_3_]/[NH^+^_4_]≈ 0.08). Even though luminal [NH_3_] was ∼100-fold higher than basolateral [NH_3_], the basolateral exposure produced an easily discernable series of pH_i_ changes, where the luminal exposure was without effect. Thus, the apical membranes of colonic crypts, like those of gastric glands, also exposed to an inhospitable environment, have no detectable permeability to either NH_3_ or NH^+^_4_. Although not part of that study, Singh also examined in three experiments the effect of luminal CO_2_/HCO^−^_3_; he found no evidence of apical CO_2_ permeability.

**The plasma membrane of *Xenopus* oocytes.** In the early 1990s, both Burckhardt & Frömter (1992) and Keicher & Meech (1994) observed that large-diameter oocytes from *Xenopus laevis*, exposed to 20 mm extracellular NH_3_/NH^+^_4_, exhibit a paradoxical fall in pH_i_, like that first reported on other cell types by Aickin & Thomas (1977) and by Kikeri *et al.* (1989). Later, Bakouh *et al.* (2006) reported that although an exposure to 10 mm NH_3_/NH^+^_4_ caused oocyte pH_i_ to fall, an exposure to 0.5 mm NH_3_/NH^+^_4_ elicited no change in pH_i_. As discussed below (section ‘*NH_3_ handling by oocytes*’), work by Musa-Aziz *et al.* (2009*c*) using surface-pH electrodes suggests that oocytes indeed have a modest permeability to NH_3_ but that the oocytes remove the incoming NH_3_ from the cytosol by either metabolism or sequestration.

As demonstrated by Preston *et al.* (1992), *Xenopus* oocytes have a relatively low osmotic water permeability (*P*_f_) except when expressing a water channel such as AQP1. From a teleological perspective, the low *P*_f_ of native oocytes is not surprising, inasmuch as amphibian blood plasma has an osmolality of ∼200 mosmol kg^−1^, whereas female *Xenopus* lay their eggs in fresh water. Thus, to the extent that water can enter *Xenopus* oocytes by osmosis, the oocytes have a tendency to swell and ultimately burst, to the extent not compensated by some energy-requiring process. It is possible that membranes facing inhospitable environments (chemically inhospitable environments in the case of the lumen of gastric glands and colonic crypts, osmotically inhospitable in the case of *Xenopus* oocytes, or perhaps physically inhospitable environments in the case of erythrocytes) have robust membranes that render them poorly permeable to water and gases.

## First evidence for gas channels: AQP1

**The importance of seminars.** On 17 October 1992, Peter Agre presented an elegant seminar to the Department of Cellular and Molecular Physiology at Yale, summarizing his groundbreaking work on aquaporins. Agre had first identified what proved to be the AQP1 protein in the membranes of erythrocytes and the kidney (Denker *et al.* 1988), and cloned the cDNA from human fetal liver (Preston & Agre, 1991). I was struck by the high level of AQP1 expression in erythrocyte membranes. After that seminar, Peter Agre arranged to send us the cDNA encoding AQP1 so that we could verify an earlier observation that AQP1 was not permeable to H^+^ (indeed, it was not). Later, he was quite magnanimous in sending us cDNA encoding other AQPs as well as AQP1 mutants. His generosity was critical to our early progress inasmuch as our molecular-biological skills at the time were rudimentary!

Nearly two years later, with Agre's AQP1 cDNA safely frozen away in our laboratory, I found myself presenting our recently published gastric-gland work to the Department of Physiology at the University of Pennsylvania. After the talk, someone asked me the obvious but still unanswered question, how is it that the apical membranes of gastric-gland cells are able to exclude NH_3_ and CO_2_? I replied that the lipids of the apical membranes may have an intrinsically low gas permeability, or contain proteins or other substances that (although not quite stated this way) reduce access/egress. As we were leaving the seminar room, Paul De Weer asked me if I considered the possibility that all membranes have an intrinsically low gas permeability, but that gastric-gland basolateral membranes have ‘gas channels’; I expressed my incredulity.

Although, years later, De Weer denied any knowledge of this conversation, my mind returned to it early and often. I reasoned that if, indeed, gas channels exist, they would possess the following properties. (1) They would most probably be found in a cell whose *raison d’être* was gas transport. (2) The channel protein(s) would be present in that cell at high levels. (3) The function of the protein would either be unknown or, if known, would not comport in an obvious way with the *raison d’être* of the cell. Before long, I realized that the cDNA for a prime candidate was languishing in our freezer. Was AQP1 not only a water channel, as so beautifully demonstrated by Agre and collaborators, but possibly also a gas channel?

**Early work with CO_2_ on *Xenopus* oocytes.** As it happened, former postdoctoral fellow Nazih Nakhoul returned to my group from 1994 to 1995 for a sabbatical. He wished to extend his technical repertoire by performing electrophysiological experiments on *Xenopus* oocytes that were heterologously expressing mammalian proteins. As first demonstrated in the landmark paper by Preston *et al.* (1992), it is easy to express AQP1 in oocytes, and also to check the adequacy of expression by dropping them in deionized water and observing the osmotic swelling and, ultimately, a rather striking explosion. Therefore, Nakhoul decided to test the gas-channel hypothesis. With the help of Bruce Davis and Michael Romero, he injected oocytes either with cDNA encoding human AQP1 or with water as a control, and then add/removed CO_2_/HCO^−^_3_. In a paper published in February 1998, Nakhoul *et al*. showed that the maximal rate of CO_2_-induced acidification (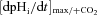), as well as the maximal rate of alkalinization induced by the removal of CO_2_ (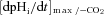), were not different in AQP1 *versus* control oocytes (Nakhoul *et al.* 1998). We reasoned that as the CO_2_ entered the cell, the reaction CO_2_+ H_2_O → H_2_CO_3_→ HCO^−^_3_+ H^+^ may have been rate limiting not only for the generation of the H^+^ that the intracellular pH electrode was measuring, but also for the clearance of CO_2_ from the inner surface of the cell membrane. Perhaps the latter effect, which would have reduced the inward gradient for CO_2_, masked any effect of AQP1.

When he injected the oocytes with carbonic anhydrase II (CAII) protein, Nakhoul found, as expected, that rates of pH_i_ change were markedly increased in all conditions (e.g. 4.8-fold for CO_2_ application in water-injected oocytes). Moreover, he found that during CO_2_ application, when pH_i_ is falling rapidly, the magnitude of the CAII-dependent component of 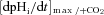 was ∼45% greater in AQP1-expressing oocytes than in control oocytes injected with water rather than cRNA. During CO_2_ withdrawal, when pH_i_ is rising rapidly, the CAII-dependent component of 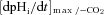 was ∼60% greater in AQP1-expressing oocytes *versus* control oocytes. Finally, the carbonic anhydrase inhibitor ethoxzolamide (ETX) erased the effect of the CAII. The magnitude of the ETX-sensitive component of 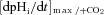 was ∼65% greater for AQP1-expressing oocytes *versus* control oocytes. Thus, these experiments proved that the heterologous expression of human AQP1 causes a significant increase in the apparent CO_2_ permeability of *Xenopus* oocytes. Although the most likely explanation was that the extra CO_2_ moved through AQP1, it was impossible to rule out, on the basis of the data alone, the possibility that the expression of AQP1 produced its effect by one of the following mechanisms: (1) increasing the background permeability of membrane lipids; (2) causing the upregulation of an unknown gas channel in oocytes; or (3) an effect of CAII on (1) or (2).

**Figure 5:**
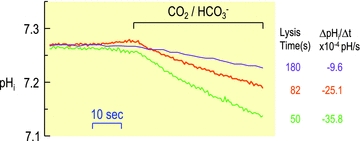
Effect of graded expression of human AQP1 on CO_2_-induced acidification rate of *Xenopus* oocytes Three oocytes (purple, orange and green records) injected with cRNA encoding human AQP1 were superfused with physiological saline at pH 7.5. Intracellular pH was monitored by impaling the cell with a liquid-membrane pH microelectrode and a conventional electrode for monitoring membrane potential. Data are from Cooper & Boron (1998). During the indicated periods, the extracellular solution was switched to one equilibrated with 1.5% CO_2_/10 mm HCO^−^_3_. The initial rate of pH_i_ decline is an index of the CO_2_ permeability. After the electrophysiological recordings, the oocytes were dropped into deionized water and monitored for the time to lysis (shorter times correlating with greater osmotic water permeabilities). Together with other data, these observations showed that CO_2_ can move through AQP1.

**Later work with CO_2_ on oocytes.** In a paper published in December 1998, Gordon Cooper found that, in oocytes lacking exogenous CAII, the expression of AQP1 had no effect on the maximal rate of CO_2_-induced acidification (Cooper & Boron, 1998), confirming the earlier work of Nakhoul *et al.* (1998). However, he found that when he removed the vitelline membrane (a manoeuvre expected to decrease the extracellular unstirred layer and thus better reveal the contribution of the cell membrane) the expression of AQP1 did indeed cause an increase in 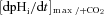. Figure 5 shows pH_i_ records from three oocytes, previously injected with cRNA encoding AQP1, and expressing this AQP1 to varying degrees, exposed to 1.5% CO_2_/10 mm HCO^−^_3_ at pH 7.50. The trace coloured purple represents the oocyte that acidified most slowly (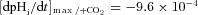 pH units s^–1^) and, when subsequently exposed to deionized water, lysed in 180 s. Neither of these values is very different from those of water-injected control oocytes. That is, this particular oocyte, which had a low level of AQP1 expression, also had an unremarkable CO_2_ permeability. The orange trace is from an oocyte that had both an intermediate acidification rate (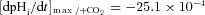 pH units s^–1^) and an intermediate lysis time (82 s). Finally, the green record is from an oocyte that acidified rapidly (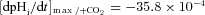 pH units s^–1^) and lysed quickly (50 s). A more extensive analysis of 34 devitellinized oocytes injected with cRNA encoding AQP1 demonstrated a decreasing linear relationship between the magnitude of 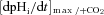 and the lysis time. In contrast, expression of the K^+^ channel ROMK1 had no effect on 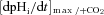 but did hyperpolarize the oocyte to the predicted equilibrium potential for potassium (*E*_K_). Thus, Cooper demonstrated that CO_2_ permeability correlates with the expression of AQP1 but not an unrelated K^+^ channel.

Macey (1984) showed that mercurials reduce the permeability of the putative water channel in red blood cells (RBCs). In 1992, Preston and colleagues demonstrated that HgCl_2_ also reduces the water permeability of AQP1 as expressed in oocytes (Preston *et al.* 1992), and in 1993 they demonstrated that Cys-189 (near the opening of the extracellular side of the water pore) is necessary for mercurial sensitivity (Preston *et al.* 1993). Therefore, Gordon Cooper examined the effect of *p*-chloromercuribenzenesulfonate (pCMBS) on the CO_2_-induced acidification. He found that pCMBS produces a larger reduction of the magnitude of 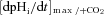 in AQP1-expressing oocytes than in water-injected control cells, and that this effect is abrogated by a mutation that converts Cys-189 to Ser (i.e. C189S). Thus, a mercurial derivative reduces the AQP1-dependent component of CO_2_ permeability, and the predicted mutation of AQP1 prevents the inhibitory effect. These results prove that AQP1 *per se* can mediate CO_2_ transport.

**Work with CO_2_ on erythrocytes.** In December 1998, Forster *et al.* (1998) made the surprising observation that DIDS not only reduces the HCO^−^_3_ permeability of RBCs (due to blockade of the Cl^−^–HCO^−^_3_ exchanger AE1), but the CO_2_ permeability as well. The experimental approach was to use ^18^O-labelled HCO^−^_3_ and use mass spectrometry to monitor the degree to which carbonic anhydrase (present only inside RBCs) accelerates the loss of the ^18^O label to H_2_O. They hypothesized that DIDS could reduce CO_2_ permeability by reacting either with the membrane lipid or with a major membrane protein, such as AE1 or AQP1. Citing an abstract by Cooper (a report that DIDS inhibited AQP1 expressed in oocytes; Boron & Cooper, 1998), Forster *et al*. favoured the membrane-protein option.

**Work with CO_2_ on reconstituted AQP1.** Finally, also in December 1998, Prasad and colleagues demonstrated that human AQP1 reconstituted into *E. coli* phospholipid vesicles increased CO_2_ permeability to about threefold above background (Prasad *et al.* 1998). Mercury chloride blocked this increase in CO_2_ permeability, and β-mercaptoethanol reversed the blockade. More recently, the senior author of that paper seems to have distanced himself from the conclusion that CO_2_ moves through AQP1 (Missner *et al.* 2008*b*).

**Work with nitric oxide.** Herrera and colleagues (Herrera *et al.* 2006; Herrera & Garvin, 2007) demonstrated that AQP1 can also transport nitric oxide (NO). Moreover, they provided evidence that AQP1-mediated NO efflux from vascular endothelial cells, as well as AQP1-mediated NO influx into smooth-muscle cells, contributes to the full effect of endothelium-dependent vasorelaxation.

## A second family of gas channels: the Rhesus (Rh) proteins

The first indication of a biological role of Rh proteins, namely, in facilitating the uptake of ‘nitrogen’, came from the observation that *Amt* (‘ammonium transporter’ in *E. coli*) and *Mep* (‘methylammonium permease’ in *Saccharomyces cerevisiae*) are essential for growth of microorganisms on a medium with NH_4_Cl as the sole nitrogen source (Fabiny *et al.* 1991; Marini *et al.* 1994). Marini *et al.* (1997) recognized that the mammalian Rh proteins are homologous to *Mep* and *Amt* in yeast, bacteria and simple plants. They also showed that transfecting *Mep*-deficient yeast with human Rh proteins restored growth in a medium containing low ammonium (Marini *et al.* 2000). Following these critical advances, functional studies led to some discussion about whether the transported species is NH_3_, NH^+^_4_, or both (see Bakouh *et al.* 2006). A key development in 2004 was the near-simultaneous determination by two groups of the X-ray crystal structure of the bacterial AmtB, which proved to be a homotrimer (Khademi *et al.* 2004; Zheng *et al.* 2004). The structural data strongly suggested that it is NH_3_, not NH^+^_4_, that passes through the pore in each of the three AmtB monomers. Crystal structures are now also available for the AmtB–GlnK complex (Conroy *et al.* 2007), the fungal Amt-1 (Andrade *et al.* 2005), the bacterial Rh50 (Lupo *et al.* 2007) and the human RhCG (Gruswitz *et al.* 2010).

Mammal Rh proteins include three erythroid proteins (RhAG, RhCE and RhD) and two non-erythroid proteins (RhBG and RhCG). Like the invertebrate Rh homologues, human RhCG is a homotrimer (Gruswitz *et al.* 2010). Moreover, an analysis of the crystal structure of RhCG, as well as of the homology of the proteins, has led to the prediction that erythroid Rh complexes are likely to be based on a template of an RhAG homotrimer, with contributions from RhCE and RhD (Gruswitz *et al.* 2010), thereby generating the experimentally determined macroscopic ratio of about 2 RhAG: 1 RhCE: 1 RhD (Eyers *et al.* 1994).

The non-erythroid Rh proteins, RhCG and RhBG, are found in a variety of mammalian tissues, including liver, lung, stomach, gastrointestinal tract and kidney (Liu *et al.* 2001; Eladari *et al.* 2002; Quentin *et al.* 2003; Weiner & Verlander, 2003; Nakhoul & Hamm, 2004; Handlogten *et al.* 2005; Weiner, 2006; Han *et al.* 2009). In the kidney, both RhBG and RhCG are present (Liu *et al.* 2000, 2001; Marini *et al.* 2000; Eladari *et al.* 2002; Verlander *et al.* 2003; Bakouh *et al.* 2006; Ripoche *et al.* 2006; Weiner & Verlander, 2010) in both the α-intercalated cells and the principal cells of the collecting duct (CD). Here, NH_3_ secretion into the lumen (in parallel with the extrusion of H^+^ into the tubule lumen to lead to the formation of NH^+^_4_) plays an important role in urinary ‘H^+^’ excretion and thus in the control of systemic pH. While RhBG is confined to the basolateral membranes (Eladari *et al.* 2002; Quentin *et al.* 2003; Verlander *et al.* 2003), RhCG is present in both the basolateral and apical membranes (Han *et al.* 2006; Seshadri *et al.* 2006; Kim *et al.* 2009). Supporting the hypothesis that RhCG is important for NH_3_ secretion by the CD are the following observations: (1) RhCG-knockout mice cannot normally acidify the urine (Biver *et al.* 2008); and (2) a CD-specific RhCG knockout exhibits depressed basal NH^+^_4_ excretion as well as an impaired increment in NH^+^_4_ excretion in response to an acid load (Lee *et al.* 2009). A specific knockout of RhCG in only the intercalated cells of the CD produces a less severe deficit in NH^+^_4_ excretion (Lee *et al.* 2010).

The erythroid Rh complex is clinically important for blood transfusions as well as for the incompatibility that can arise between RhD-negative mothers and their RhD-positive fetuses (see Colin *et al.* 1991). The first identified function of the erythroid Rh complex was as a conduit for NH_3_ (Ripoche *et al.* 2004, 2006; Bakouh *et al.* 2006; Musa-Aziz *et al.* 2009*a*). In addition, evidence has accumulated that the Rh complex, or simply RhAG, serves as a pathway for CO_2_ (Ripoche *et al.* 2006; Endeward *et al.* 2008; Musa-Aziz *et al.* 2009*a*). It will be interesting to see whether the Rh complex conducts other gases, such as O_2_ and NO.

## Use of surface-pH measurements to study gas transport

**Figure 6:**
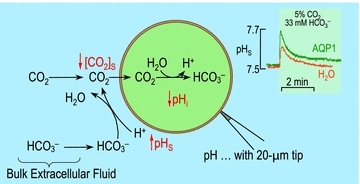
Model of surface pH (pH_S_) changes caused by the influx of CO_2_ The influx of CO_2_ not only causes a fall of pH_i_ but also a transient rise of pH_S_. The two inset pH_S_ records at the top right come from oocytes injected either with water or with cRNA encoding human AQP1, measured with liquid-membrane pH-sensitive microelectrodes that initially just touched the membrane surface and then were advanced an additional ∼40 μm. Data are from Musa-Aziz *et al.* (2009*a*).

**Background.** As part of another project, Raif Musa-Aziz was monitoring the surface pH (pH_S_) of oocytes with a polished liquid-membrane mini-electrode (tip diameter ∼20 μm) that she pushed up against the oocyte, dimpling the membrane slightly. She found that applying CO_2_/HCO^−^_3_ in the extracellular fluid causes a predictable pH_S_ transient that is similar to the pH_o_ waveform reported long before by De Hemptinne & Huguenin (1984) in their studies on skeletal muscle. As shown in the main portion of Fig. 6, the influx of CO_2_ creates, near the outer surface of the cell membrane, a decline of [CO_2_] that both provides a gradient for CO_2_ diffusion from the bulk extracellular fluid and, at the cell surface, drives the net reaction HCO^−^_3_+ H^+^→ H_2_CO_3_→ CO_2_+ H_2_O. The orange record in Fig. 6, for a water-injected oocyte, shows that introducing CO_2_/HCO^−^_3_ causes pH_S_ to rise abruptly to a peak that presumably coincides with the maximal rate of CO_2_ entry (Musa-Aziz *et al.* 2009*a*). We define the maximal magnitude of this peak as ΔpH_S_. The slow pH_S_ decay occurs as CO_2_ equilibrates across the membrane (in Fig. 2 we saw the pH_i_ consequences of such a slow CO_2_ equilibration). The green trace in Fig. 6 shows similar results for an oocyte expressing AQP1. Since the ΔpH_S_ spike reflects the maximal CO_2_ influx, these experiments confirm that AQP1 serves as a conduit for CO_2_.

**Figure 7:**
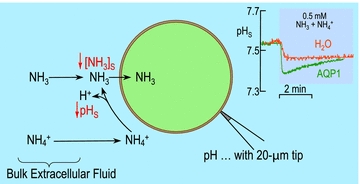
Model of pH_S_ changes caused by the influx of NH_3_ The influx of NH_3_ not only causes a rise of pH_i_ but also a transient fall of pH_S_. The two inset pH_S_ records at the top right come from oocytes injected either with water or with cRNA encoding human AQP1; the same oocytes as in Fig. 6. Data are from Musa-Aziz *et al.* (2009*a*).

Exposing a cell to NH_3_/NH^+^_4_ causes an opposite series of pH_S_ changes, as first observed by Chesler in his pH_o_ measurements of lamprey neurons (Chesler, 1986). As illustrated in the main portion of Fig. 7, the influx of NH_3_ triggers a decline of [NH_3_]_S_ that both drives NH_3_ diffusion from the bulk extracellular fluid and, at the cell surface, drives the net reaction NH_3_+ H^+^→ NH^+^_4_. The orange record in Fig. 7, for a water-injected oocyte, shows that introducing NH_3_/NH^+^_4_ causes pH_S_ to fall abruptly to a nadir that presumably coincides with the maximal rate of NH_3_ entry (Musa-Aziz *et al.* 2009*a*). The green trace in Fig. 7 shows similar results for an oocyte expressing AQP1, and confirms that AQP1 also provides a pathway for NH_3_.

These experiments show that it is rather easy to extract from pH_S_ transients a semi-quantitative index of maximal CO_2_ or NH_3_ flux, which translates to a semi-quantitative index of macroscopic permeability. In principle, this pH_S_ approach could work with any neutral weak acid or base. Indeed, when Musa-Aziz and colleagues (Musa-Aziz *et al.* 2009*a*) exposed oocytes to butyrate/butyric acid, they observed pH_S_ transients like those triggered by CO_2_ in Fig. 6, except that AQP1 did not enhance permeability to butyric acid. However, by way of caution, I point out that it will not be trivial to extract the membrane permeability to CO_2_ or butyric acid or NH_3_ from pH_S_ transients. Colleagues at my home institution (Daniela Calvetti and Erkki Somersalo from the Department of Mathematics, as well as Rossana Occhipinti, who joined our group after completing her PhD with the Calvetti-Somersalo group) have modelled the system as a spherical cell in which reaction and diffusion processes occur simultaneously. The model seems to be reasonable from the perspective of pH_i_ measurements. However, it is clear that the pH_S_ electrode creates a special environment that accentuates pH_S_ transients and that more modelling will be required for a quantitative understanding of the physiology within this special environment.

**Handling of NH_3_ by oocytes.** I have already noted that the plasma membrane of *Xenopus* oocytes is unusual (see section ‘*The plasma membrane of Xenopus oocytes*’), with oocytes responding to the application of high NH_3_/NH^+^_4_ levels (e.g. 10–20 mm) with a paradoxical fall in pH_i_, but to low NH_3_/NH^+^_4_ levels with little change in pH_i_. Musa-Aziz and colleagues re-examined this issue using, in addition to pH_i_, both pH_S_ and NMR methods, and the new data led to the following conclusions, which are quite surprising (Musa-Aziz *et al.* 2009*c*). (1) Regardless of whether [NH_3_/NH^+^_4_]_o_ is high or low, and regardless of the presence *versus* the absence of the bacterial Rh homologue AmtB, the influx of NH_3_ (rather than the influx of NH^+^_4_) dominates pH_S_ and would dominate pH_i_ if other factors did not come into play. (2) For these and other reasons discussed, the paradoxical fall in pH_i_ observed at high [NH_3_/NH^+^_4_]_o_ cannot be due to the influx of NH^+^_4_. The pH_i_ decrease could result from the triggered production of intracellular H^+^. (3) AmtB enhances the influx of NH_3_ over that of NH^+^_4_. (4) Once it has entered the oocyte, nearly all NH_3_ appears to be sequestered as NH^+^_4_, presumably in acidic compartments. (5) The removal of extracellular NH_3_/NH^+^_4_ merely terminates, for the most part, the influx of NH_3_; it does not, over the period of our observation, produce a large, symmetrical efflux of NH_3_. (6) A hypothetical, extracellular, low-affinity sensor for NH_3_ or NH^+^_4_ (perhaps an adaptation that allows oocytes to survive in pond water that contains decaying organic matter) could trigger the aforementioned production of intracellular H^+^.

**Gas selectivity.** Armed with the pH_S_ approach summarized in Figs 6 and 7, Musa-Aziz and colleagues embarked on a series of experiments in which they injected oocytes with either water or cRNA encoding AQP1 (expressed at high levels in RBCs), AQP4 (highly expressed in the blood–brain barrier), AQP5 (highly expressed in alveolar type I pneumocytes), AmtB, RhAG (RBCs) or other membrane proteins. Later, they sequentially measured in each oocyte the ΔpH_S_ evoked by CO_2_/HCO^−^_3_, the ΔpH_S_ evoked by NH_3_/NH^+^_4_ and the osmotic water permeability. By comparing the data from oocytes expressing channels with data from day-matched water-injected control cells, they were able to obtain the following channel-dependent values (designated by*) for each oocyte: 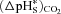, 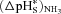 and P*_f_.

**Figure 8:**
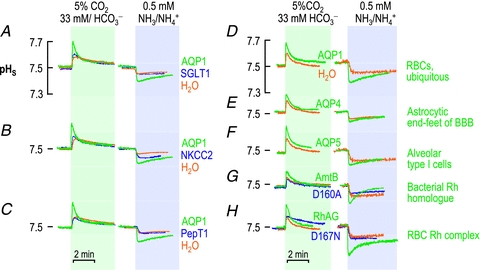
Paired pH_S_ transients in single oocytes caused by the influx of CO_2_ and then the influx of NH_3_ Oocytes were injected with either water or cRNA encoding the indicated membrane protein, and then superfused with physiological saline at pH 7.5. The pH_S_ was monitored as outlined in Figs 6 and 7. For each oocyte, CO_2_/HCO^−^_3_ was introduced (left half of each panel), washed out (not shown), and then NH_3_/NH^+^_4_ was introduced (right half of each panel). *A–C* show that AQP1 but not three transporters can support the pH_S_ transients. *D–H* show that AQP1, AQP4, AQP5, AmtB and RhAG can each transport CO_2_, but only AQP1, AmtB and RhAG can transport NH_3_. Data are from Musa-Aziz *et al.* (2009*a*).

The eight panels in Fig. 8 show representative examples of CO_2_- and NH_3_-evoked pH_S_ transients for oocytes expressing various membrane proteins. In each case, we first exposed the oocyte to 5% CO_2_/33 mm HCO^−^_3_ at a fixed pH_o_ of 7.50 (left side of panel), then removed the CO_2_/HCO^−^_3_ (not shown), and then exposed the same oocyte to 0.5 mm NH_3_/NH^+^_4_ at pH_o_ 7.50 (right side of panel). In each of the first three panels (Fig. 8*A*–*C*), we show three records (obtained on the same day from a single batch of oocytes), one from a water-injected control oocyte (the same one in each panel), one from an oocyte expressing AQP1 (again, the same oocyte in each panel) and one from an oocyte expressing the Na^+^–glucose cotransporter SGLT1 (Fig. 8*A*) or the Na^+^–K^+^–2Cl^−^ cotransporter NKCC2 (Fig. 8*B*) or the H^+^–oligopeptide cotransporter PepT1 (Fig. 8*C*). For each of the three cotransporter oocytes, the pH_S_ record is indistinguishable from that of the water-injected control oocyte, and exhibits a ΔpH_S_ that is substantially less than the AQP1 oocyte. Mean data from the larger study confirm this conclusion. Thus, not every membrane protein is a gas channel.

The next group of five panels (Fig. 8*D*–*H*) shows the results of experiments on five putative gas channels, each compared with a day-matched water-injected control oocyte. Mean data from the larger study confirm the impression conveyed by these five panels. In response to the application of CO_2_/HCO^−^_3_, all five wild-type channels produce ΔpH_S_ values that are significantly larger than those of their day-matched controls. Likewise, in response to the application of NH_3_/NH^+^_4_, AQP1, AmtB and RhAG all produce ΔpH_S_ values that are significantly larger than those of their day-matched controls. However, AQP4 (Fig. 8*E*) and AQP5 (Fig. 8*F*) show no significant permeability to NH_3_. Also, mutations to AmtB (Fig. 8*G*) and RhAG (Fig. 8*H*) that are known to render them inactive also reduce 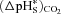 and 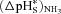 to values that are indistinguishable from those of water-injected oocytes.

**Figure 9:**
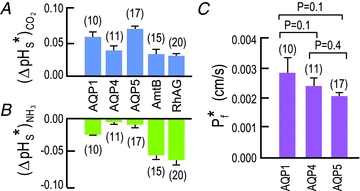
Mean channel-dependent changes in maximal rise of pH_S_ caused by CO_2_ influx (*A*), maximal fall of pH_S_ caused by NH_3_ influx (*B*) and osmotic water permeability (*C*) The semi-quantitative index of maximal CO_2_ flux,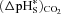, is the maximal rise in pH_S_ (ΔpH_S_) in oocytes expressing a channel, less the mean ΔpH_S_ of day-matched control oocytes (i.e. water-injected oocytes). The semi-quantitative index of maximal NH_3_ flux, 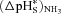, is the greatest extent of the fall in pH_S_ (ΔpH_S_) in oocytes expressing a channel, less the mean ΔpH_S_ of day-matched control oocytes (i.e. water-injected oocytes). The value of 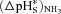 is not significantly different from zero for either AQP4 or AQP5. *P*_f_* is the analogous figure for osmotic water permeability. Note that neither AmtB nor RhAG significantly conducted water. Data are from Musa-Aziz *et al.* (2009*a*).

Because each oocyte yielded values for 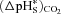, 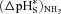 and P*_f_, it is possible to get a sense of the relative permeability of each channel to each substance. Figure 9 summarizes the mean channel-specific ΔpH_S_ values for CO_2_ (Fig. 9*A*) and NH_3_ (Fig. 9*B*) as well as the channel-specific *P*_f_ values (Fig. 9*C*). Note that neither AmtB nor RhAG had any significant water permeability (not shown).

**Figure 10:**
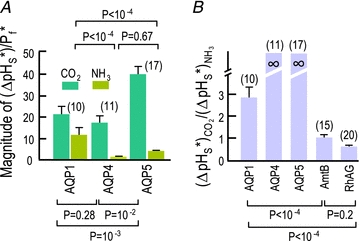
Mean values of
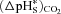
**and**
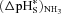
normalized to *P*_f_* (*A*) and mean values of
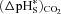
**normalized to**
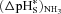
**(*B*)** The values in *A* were obtained by dividing the values of 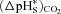 and 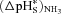 for each oocyte by the value of *P*_f_*. These ratios are semi-quantitative indices of CO_2_/H_2_O permeability ratios and the NH_3_/H_2_O permeability ratios. The values in *B* were obtained by dividing the values of 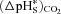 for each oocyte by the value of 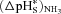. These ratios are semi-quantitative indices of CO_2_/NH_3_ permeability ratios. Since 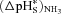 was not statistically different from zero for AQP4 and AQP5, the NH_3_/H_2_O ratios for these channels should not be different from zero. Likewise, the CO_2_/NH_3_ ratios are theoretically infinite. Data are from Musa-Aziz *et al.* (2009*a*).

If we now, oocyte by oocyte, divide the 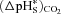 value that contributes to Fig. 9*A* by the P*_f_ value that contributes to Fig. 9*C*, we arrive at a relative index of CO_2_, normalized to the H_2_O permeability of the three AQPs (turquoise bars in Fig. 10*A*). We see that AQP5 has the highest CO_2_/H_2_O permeability ratio by about a factor of two, with AQP1 and AQP4 following.

Likewise, if we divide the 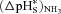 value that contributes to Fig. 9*B* by the P*_f_ value that contributes to Fig. 9*C*, we arrive at a relative index of NH_3_*versus* H_2_O permeability for the three AQPs (pea-green bars in Fig. 10*A*). We see that AQP1 has the highest NH_3_/H_2_O permeability ratio; those for AQP4 and AQP5 are essentially zero because neither has a statistically significant NH_3_ permeability. At the other extreme, AmtB and RhAG, for which the H_2_O permeability is negligible, have NH_3_/H_2_O ratios approaching infinity.

Finally, if we divide the 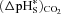 value that contributes to Fig. 9*A* by the 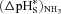 value that contributes to Fig. 9*B*, we arrive at a relative index of CO_2_*versus* NH_3_ permeability for all five channels (Fig. 10*B*). Since the mean 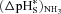 values for AQP4 and AQP5 do not statistically differ from zero, their ratios are essentially infinity, followed by AQP1, which has a ratio about threefold greater than for AmtB, which in turn has a ratio about twice that of RhAG.

Note that 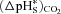 and 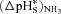 are each semi-quantitative indices of permeability, not permeabilities themselves. Thus, the ratios in Fig. 10 are relative indices of channel permeability, not permeability ratios *per se*. Nevertheless, we obtained all values (and continue to obtain values on other AQPs and Rh proteins) in standard conditions, so that it is meaningful to compare values for the different channels. Ongoing mathematical modelling may eventually yield estimates of absolute permeabilities from pH_S_ data.

**Mechanism of CO_2_ and NH_3_ permeation through the AQPs and Rh proteins.** The data in Figs 8–10 represent the first demonstration of gas selectivity by membrane proteins. An obvious and important question is, ‘What is the molecular basis for this selectivity?’ Obviously, the size of the transported substance, relative to the size of the pore through which it travels, must be important. However, chemistry must also play a key role. Recall that H_2_O and NH_3_ have similar dipole moments and that both have tetrahedral electronic structures (compared with H_2_O, NH_3_ has a proton in place of one lone pair of electrons). Thus, we should not be surprised if sometimes H_2_O and NH_3_ behave in a similar manner. Carbon dioxide, in contrast, is a linear molecule (O=C=O) with no dipole moment but a quadrupole moment, due to residual negative charge at each oxygen. Thus, CO_2_ is much less hydrophilic than H_2_O or NH_3_. Oxygen, which has no charge separation, is far more hydrophobic.

Regarding the chemistry of the proteins, X-ray structures show that the four monomeric aquapores of AQP1 (Murata *et al.* 2000; Sui *et al.* 2001), for example, have both hydrophilic and hydrophobic surfaces. However, the central pore at the fourfold axis of symmetry is mainly hydrophobic. Molecular dynamic simulations (Tajkhorshid *et al.* 2002) suggest that the H_2_O molecules move single file through the aquapore, backing into the pore oxygen-first, flipping orientation near the overlapping asparagine-proline-alanine (NPA) motifs at the centre of the aquapore, and then emerging oxygen-last from the pore. Other molecular dynamic simulations suggest that CO_2_ can move single file through an aquapore, interposed between H_2_O molecules (Wang *et al.* 2007). However, these simulations predict that CO_2_, and particularly O_2_, would move far more readily through the central pore. Since, in the absence of dissolved gases, this central pore is predicted not only to be large enough to accept CO_2_ but also to be empty (i.e. a vacuum), the central pore could be a highly efficient gas channel. Note that the mobility of CO_2_ in the gas phase (∼1.0 × 10^−1^ cm^2^ s^−1^ at 20°C; see Weast, 1978) is about four orders of magnitude greater than through water (∼1.8 × 10^−5^ cm^2^ s^−1^ at 20°C; see Tamimi *et al.* 1994).

The three monomeric NH_3_ pores of a homotrimer in the Rh family have a generally hydrophobic character (Khademi *et al.* 2004; Zheng *et al.* 2004; Khademi & Stroud, 2006; Gruswitz *et al.* 2010), but with a conserved antiparallel pair of His residues at the centre. A peculiarity is that the openings to the NH_3_ pores are guarded by residues that apparently attract NH^+^_4_. Thus, the hypothesized mechanism of transport is that an NH^+^_4_ ion from the bulk solution approaches the mouth of the pore and dissociates. The H^+^ would diffuse back into the bulk solution, whereas only the NH_3_ would enter the predominantly hydrophobic NH_3_ pore. Upon exiting from the opposite end of the pore, the NH_3_ would combine with an H^+^ ion (which would diffuse in from the bulk fluid), and the nitrogen would diffuse into the bulk fluid as NH^+^_4_.

We have been gaining some insight into the mechanism of gas transport by using inhibitors. Preliminary work by Musa-Aziz and colleagues on AQP1 suggests that the mercurial pCMBS, which is known to inhibit H_2_O transport, also reduces 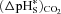 by ∼40% and eliminates NH_3_ permeability (Musa-Aziz *et al.* 2007*a*,*b*, 2008, 2009*b*). The C189S mutant of AQP1 is immune to these effects of pCMBS. Also with AQP1, we find that DIDS has no effect on either H_2_O or NH_3_ permeability, but reduces 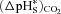 by ∼60%. These effects of DIDS persist after scavenging with albumin, consistent with the idea that the DIDS reacts covalently with the AQP1. The DIDS blockade is also unaffected by the C189S mutation. The combination of pCMBS and DIDS reduces 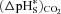 by ∼100%. Thus, the two inhibitors act on separate pathways that, together, account for all of the CO_2_ permeability of AQP1. One pathway, accounting for all H_2_O and NH_3_ transport, and ∼40% of the CO_2_, is the monomeric aquapore. Although the other pathway is yet to be established, a reasonable candidate is the central pore.

In the case of AQP4, preliminary data from Musa-Aziz *et al.* (2007*b*) suggest that DIDS blocks nearly all CO_2_ permeability, but again none of the H_2_O permeability. In the case of AQP5, preliminary work by Musa-Aziz *et al.* (2007*b*) and by Qin & Boron (2010) suggests that DIDS blocks ∼75% of the CO_2_ permeability, but none of the H_2_O permeability. Thus, it is reasonable to suggest that nearly all CO_2_, and perhaps O_2_ as well, moves through an alternative pathway of these AQPs, perhaps the central pore.

In the cases of AmtB and RhAG, we find that the H_2_O permeability is zero (Musa-Aziz *et al.* 2009*a*). Moreover, preliminary work from Musa-Aziz shows that DIDS has no effect on the NH_3_ permeability, but blocks virtually all CO_2_ permeability. Thus, like the AQPs, the Rh proteins seem to have two distinct pathways for gas transport. One pathway is the monomeric NH_3_ pore that conducts NH_3_ but apparently not H_2_O or substantial amounts of CO_2_. The other pathway is the conduit for CO_2_, and could be the central pore of the Rh proteins. It will be interesting to see whether O_2_ moves through the Rh proteins and, if so, whether it follows the same path as CO_2_.

## Possible physiological significance of AQPs as gas channels

The first report of a possible physiological role for an AQP as a gas channel came from Uehlein *et al.* (2003), who reported that an AQP in tobacco plants functions as a CO_2_ channel and promotes photosynthesis and plant growth.

**Roles of AQP1 and Rh complex in RBCs.** After the Cl^−^–HCO^−^_3_ exchanger AE1, the second and third most abundant integral membrane proteins in the mammalian erythrocyte are AQP1 and the Rh complex. In 2006, Endeward and colleagues reported work in which they used ^18^O-labelled HCO^−^_3_ to study the CO_2_ permeability of wild-type (WT) *versus* AQP1-null human RBCs (Endeward *et al.* 2006). They found that CO_2_ permeability was reduced by ∼60% in the AQP1-null RBCs, and that these cells were insensitive to pCMBS. The combination of the absence of AQP1 and the presence of DIDS (which we now appreciate, as noted above, blocks the remnant CO_2_ permeability mediated by the Rh complex) reduced CO_2_ permeability by ∼95%. Thus, at most 5% of the CO_2_ could move through the lipid of the plasma membrane.

In 2008, Endeward and colleagues published the complementary work on Rh-null human RBCs (Endeward *et al.* 2008). They found that the absence of the Rh complex reduced the CO_2_ permeability by nearly half. This observation supports the conclusion from the previous paragraph; nearly all CO_2_ movement through the RBC membrane is mediated by either AE1 or the Rh complex.

In the pulmonary capillary bed, CO_2_ comes to diffusion equilibrium between the blood and the alveolar air about one-third of the way along the pulmonary capillary. It is possible that, with the increase in cardiac output that accompanies maximal exercise, the contact time of RBCs with pulmonary capillaries would be sufficiently reduced as to decrease the offloading of CO_2_ in the absence of AQP1 and/or the Rh complex, resulting in metabolic acidosis. In principle, the body could compensate by increasing alveolar ventilation, though at the cost of increased work. Another potential role of the channels in RBCs would be as conduits for O_2_. During exercise, the absence of the gas channels could lead to a net reduction in O_2_ uptake by the end of the pulmonary capillary, causing arterial hypoxaemia, which in turn could limit aerobic exercise.

**Effect of AQP1 knockout on exercise.** In preliminary work by Xu *et al.* (2010), we have examined voluntary exercise on activity wheels in WT and AQP1-null mice. Wild-type mice that have never seen a wheel typically run 10–12 km day^−1^ in the absence of any resistance on the wheel. Over a wide range of ambient O_2_ levels in the absence of resistance, the distance run by knockout mice is reduced by ∼40% compared with WT mice.

Key unanswered questions are whether this exercise deficit is partly overcome by raising ambient [O_2_], and whether AQP1 specifically in RBCs plays a role.

**Possible role of AQP1 in zebrafish swimbladder.** We recently cloned AQP1a from zebrafish (Chen *et al.* 2010), finding that the protein is most highly expressed in RBCs, the swimbladder, and in regions of the avascular retina that correspond to the portions of the photoreceptor cell that contains mitochondria.

During the period immediately after making the transition from embryo to larva, the zebrafish has poorly developed gills, and inflates its swimbladder (connected to the oesophagus by a pneumatic duct) by gulping air. In unpublished work, Nick Courtney finds that if he replaces room air with 100% N_2_ between 3 and 10 days postfertilization, then at day 10, the ‘100% N_2_’ fish have a dry mass that is ∼25% less than their room-air littermates. These data are consistent with the hypothesis that, at least during this part of the zebrafish life, the swimbladder functions as a respiratory organ. We are extending these experiments to several combinations of [O_2_] in the water and gas phases, and hope to be able to repeat the work with AQP1a-null zebrafish.

**Figure 11:**
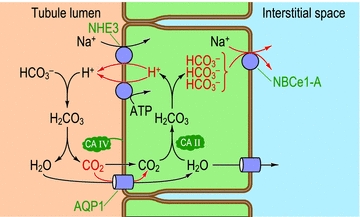
Model of HCO^−^_3_ reabsorption by the renal proximal tubule Bicarbonate appears in the lumen of the proximal tubule as the result of glomerular filtration. Abbreviations: AQP1, aquaporin 1; CAII, carbonic anhydrase II; CAIV, carbonic anhydrase IV; NBCe1-A, renal splice variant of electrogenic Na/HCO_3_ cotransporter 1; and NHE3, Na^+^–H^+^ exchanger 3.

**Role of AQP1 in HCO^−^_3_ reabsorption by the renal proximal tubule.** One of the major tasks of the renal proximal tubule (PT) is to reabsorb (i.e. to move from lumen to blood) ∼80% of the HCO^−^_3_ filtered at the glomerulus. More distal portions of the nephron reabsorb the remainder of the filtered HCO^−^_3_. As outlined in Fig. 11, the cells of the PT secrete H^+^ into the tubule lumen using both the Na^+^–H^+^ exchanger NHE3 and a vacuolar-type H^+^ pump at the apical membrane. Once in the lumen, the H^+^ titrates HCO^−^_3_ to form H_2_O and CO_2_, catalysed by carbonic anhydrase IV (CAIV), which is linked to the apical membrane. The secreted H^+^ also titrates weak bases other than HCO^−^_3_, such as NH_3_ and inorganic phosphate. The titration of these other weak bases removes from the body the H^+^ that accumulates in the body as the result of metabolism and the ingestion of acidic foodstuffs. The newly formed CO_2_ and H_2_O enter the cell, where they recombine to form H^+^ and HCO^−^_3_, catalysed by the soluble enzyme CAII. The cell exports the H^+^ across the apical membrane to the lumen as noted above, and uses the electrogenic Na^+^–HCO^−^_3_ cotransporter NBCe1-A to move the HCO^−^_3_ across the basolateral membrane and into the interstitial space, which is in contact with the blood.

Let us return now to the H_2_O and CO_2_ formed in the lumen. The vast majority of the reabsorbed H_2_O moves across the apical and basolateral membranes through AQP1 (Schnermann *et al.* 1998; Vallon *et al.* 2000). Preliminary data by Zhou *et al.* (2006) show that the maximal HCO^−^_3_ reabsorption rate is reduced by ∼60% in PTs from AQP1-null mice compared with WT mice. Alan Verkman generously provided the AQP1-null mice. In additional experiments, Zhao *et al.* (1995) perfused the tubule lumen with a CO_2_/HCO^−^_3_-free solution and then used out-of-equilibrium solution technology to present to the basolateral solution either HCO^−^_3_ in the absence of CO_2_ or CO_2_ in the absence of HCO^−^_3_, always at pH 7.40. They found that with only HCO^−^_3_ in the bath, the ‘carbon backflux’ from bath to lumen was identical in AQP1-null *versus* WT tubules (Zhou *et al.* 2006). However, with only CO_2_ in the bath, the ‘carbon backflux’ from bath to lumen was ∼60% lower in AQP1-null *versus* WT tubules. Thus, AQP1 seems to be required for ∼60% of the transepithelial CO_2_ permeability of the PT.

If this AQP1-dependent CO_2_ permeability is physiologically important, we might predict that AQP1-null mice distal nephron segments, in the absence of a challenge to their acid–base status, would compensate for the deficit in PT function, and that the AQP1-null mice would have a more-or-less normal arterial pH. However, we might also predict that in the face of a chronic metabolic or respiratory acidosis, the AQP1-null mice would be unable to adapt further, and thus would exhibit a low arterial pH relative to WT mice.

## Scrutiny

The gas-channel hypothesis, if correct and if it proves to be physiologically important, would represent a major paradigm shift. Thus, it is healthy that this emerging paradigm be held up to close examination.

**A view from stopped-flow and mice.** An early analysis of the gas-channel hypothesis revolved around the following three types of experiments (Yang *et al.* 2000; Fang *et al.* 2002): (1) stopped-flow analysis of WT *versus* AQP1-null RBCs; (2) stopped-flow analysis of liposomes with or without reconstituted AQP1; and (3) the uptake of CO_2_ by artificially ventilated lungs of WT *versus* AQP1-null mice. As previously discussed (Cooper *et al.* 2002), the RBC stopped-flow experiments yielded values for CO_2_ permeability that were at least one order of magnitude smaller than those of earlier workers, and was probably due to inadequate mixing in the stopped-flow apparatus, generating large unstirred layers. This same limitation probably applied to the liposomes. In both cases, it would have been difficult to detect the CO_2_ permeability of AQP1. In the mouse-lung experiments (no doubt a technical feat), the introduction of CO_2_ into the inspired air led to the expected increase in arterial partial pressure of CO_2_. However, the half-time for the rise in arterial CO_2_ partial pressure (∼2 min) was far slower than what we would have expected for the wash-in of CO_2_ into the alveoli. Thus, it would have been difficult to detect an effect of AQP1 on CO_2_ permeability.

**A view from artificial bilayers.** More recently, in a series of physical chemistry papers by Missner and colleagues (Missner *et al.* 2008*a*,*b*; Missner & Pohl, 2009), the authors, who studied artificial planar lipid bilayers, concluded that ‘Overton continues to rule’. Their twofold argument, in brief, is as follows. (1) The unstirred layers enveloping a membrane are so large that their aggregate resistance dominates the macroscopic resistance to the diffusion of a substance such as CO_2_ from one bulk aqueous solution, through the membrane, to another bulk aqueous solution. Stated somewhat differently, the resistance offered by the membrane is simply too small to be significant. (2) The membrane lipid has such a high gas (e.g. CO_2_) permeability that the presence of a protein channel could not enhance the flux. My sense is that the aforementioned experimental work of Missner and colleagues is basically correct, as are the conclusions that narrowly flow from that work. Where we differ is on the application of general principles to real biological membranes; problems of series and parallel resistances.

We have already introduced the importance of unstirred layers, which represent a resistance to diffusion in series with the membrane lipid (see section ‘*Overton's rule*’). If one were to set up experimental conditions with increasingly large unstirred layers, it would become increasingly difficult to detect the contribution of the membrane. One could compound matters by choosing a membrane with a low baseline resistance to gas diffusion. This is the situation that prevails when working with planar artificial lipid bilayers: large unstirred layers (100–200 μm) and membranes composed only of lipids and, at that, lipids with high intrinsic permeabilities to gases such as CO_2_. In such a system, the membrane makes an insignificant contribution to macroscopic CO_2_ resistance, independent of the presence of gas channels or the validity of Overton's rule. This is the problem of the series resistance. It will be difficult for the experimenter to detect the action of gas channels unless the unstirred layers are sufficiently small relative to the resistance of the membrane with or without the channel. Since biological unstirred layers are generally tiny where they count (e.g. surrounding the RBC membrane, from alveolar air to pulmonary capillary blood), the series resistance is not a problem for physiology, but rather for physiologists trying to make measurements.

Even if one were to reduce the aggregate unstirred layer by a couple orders of magnitude to mimic the conditions faced, for example, by mammalian RBCs or the proximal-tubule apical membrane, gas channels could not enhance permeability if embedded in a sea of highly permeable lipid. Indeed, as noted by Tajkhorshid and colleagues (Wang *et al.* 2007), molecular-dynamics modelling suggests that introducing AQP1, or presumably any protein, into a membrane made of palmitoyl-oleoyl-phosphatidylethanolamine would decrease overall membrane permeability owing to the high permeability of the lipid *per se*. This is the problem of parallel resistance. A gas channel cannot enhance permeability unless the lipid surrounding the channel is relatively tight.

Thus, in order for a gas channel to enhance the permeability of a membrane to CO_2_, ignoring unstirred layers, the surrounding lipid must be exceptionally tight. I suspect that this is almost never the case in artificial systems, and it may not be the rule even in living organisms. Thus, even though Overton's rule is overly simplistic from a biophysics perspective, the classical notion that gases diffuse through the lipid phase of the membrane is probably valid for many cell membranes, but not all.

**Where might gas channels make sense?** As outlined previously (Cooper *et al.* 2002) they might make sense in the following conditions.

- When the background or intrinsic permeability of the membrane lipid is low. This is a *sine qua non*, which is why I devoted attention to the access-solubility-diffusion-protein-egress hypothesis. Several biological membranes probably fit the bill. Candidates might include any membrane that faces a physical or chemical environment that is sufficiently hostile as to require a robust membrane. Erythrocytes and *Xenopus* oocytes come to mind. Perhaps the quintessential membranes in this regard are the ones that got us thinking outside the box about gas transport in the first place, the apical membranes of gastric glands, but these certainly lack gas channels. Another fertile recruiting ground might be membranes that are required to withstand large chemical or electrical gradients. Apical membranes of certain epithelia (e.g. renal collecting ducts) and the mitochondrial inner membrane are possibilities.
- When the gas gradient is small. Examples might be the influx of CO_2_ from air (0.03% CO_2_) into plant cells, or from tissues into systemic capillaries.
- When the required gas flux is high. Examples would be the alveolar–capillary barrier in the lung, the RBC membrane and the apical membrane of proximal tubules.

The second and third bullet points merely restate eqn (5).

One conclusion to be drawn from the above analysis is that those wishing to study gas channels must use an experimental system that has a favourable combination of small unstirred layers, a low background permeability of membrane lipids and a high expression level of the channel. There are no absolutes; one can overcome the disadvantages of a somewhat larger unstirred layer if the intrinsic permeability of the membrane is sufficiently low, as seems to be the case in the *Xenopus* oocyte.

## Concluding remarks

Long before the first evidence for gas movement through channels, the discovery of water channels represented a major milestone in membrane biology. Moreover, certain members of the AQP family can, in addition to water, transport small organic molecules, such as glycerol and urea (Murata *et al.* 2000; Sui *et al.* 2001). If gas transport through the AQPs proves to be physiologically relevant, this fact would further underscore the importance of the AQP family.

One should not view H_2_O and CO_2_ movement through AQP1, AQP4 or AQP5 as an either–or issue, although one could imagine that a cell could independently gate the monomeric aquapores and the alternative CO_2_ pathway (e.g. central pore). If we will ignore this possibility for the moment, then (if gas permeation proves to be physiologically important) the major physiological contribution of an AQP would depend on its anatomical context.

At one extreme, AQP2 in the renal collecting duct, for example, might conduct CO_2_, but presumably that function would be of minor significance compared with the impact on water homeostasis.

At the other extreme, it is not clear why the H_2_O permeability *per se* of AQP1 confers an advantage in the mission of an erythrocyte, which has evolved to carry CO_2_ and O_2_ efficiently. On the contrary, it is possible that a high RBC H_2_O permeability would render the cell vulnerable to rapid shrinkage as the RBC flows deep into the hypertonic renal medulla. Perhaps this selective pressure led to the presence of urea transporters to reduce the reflection coefficient and thereby minimize volume changes.

In the middle of this spectrum might be AQP1 in the apical membrane of the renal proximal tubule. Here the AQP1 is necessary for the high H_2_O permeability that allows the PT to reabsorb large volumes of essentially isotonic saline. However, the AQP1 also appears to be responsible for 60% of the CO_2_ permeability that is necessary for HCO^−^_3_ reabsorption.

In the brain, the membrane of the astrocytic end-feet that envelop blood vessels contains semi-crystalline arrays of AQP4 that occupy about one-third of the total membrane surface area. Knocking out the AQP4 reduces the osmotic water permeability of the blood–brain barrier by ∼90% and renders the mice more resistant to the cerebral oedema that occurs following a model of stroke (Manley *et al.* 2000). Might AQP4 contribute to the CO_2_ flux across the blood–brain barrier? It is interesting to recall that AQP4 has a negligible permeability to NH_3_, which is neurotoxic.

In principle, AQP5 in the lung could serve as a pathway for CO_2_ across the apical membranes of the type I alveolar pneumocytes.

The discovery that the Rh proteins conduct NH_3_ and CO_2_ provides a function for the erythroid Rh complex that heretofore has had only a pathological role.

Finally, what advantages might gas channels provide?

- When the background gas permeability of a membrane is low, channels would enhance flux.
- Channels would allow cells to display selectivity for particular gases.
- Channels would allow cells to regulate gas permeability.
- Although not an advantage to the cell *per se*, an advantage to the scientist or physician is that gas channels could make gas permeability amenable to selective pharmacological intervention.

Thus far, the only gases of relevance to mammals that have been studied in the context of gas channels are CO_2_, NH_3_ and NO. Conspicuous by its absence from this list is O_2_. Moving forward, it will be important to make progress on the following four fronts: (1) extending the work to O_2_ as well as CO, N_2_ and other gases; (2) understanding the molecular mechanism of gas transport and selectivity by AQP and Rh channels; (3) determining whether cells can gate or otherwise regulate gas channels; and (4) testing the physiological relevance of gas transport through channels.
